# Protonic Ceramic Electrochemical Cells for Synthesizing Sustainable Chemicals and Fuels

**DOI:** 10.1002/advs.202206478

**Published:** 2023-01-18

**Authors:** Fan Liu, Dong Ding, Chuancheng Duan

**Affiliations:** ^1^ Department of Chemical Engineering Kansas State University Manhattan KS 66503 USA; ^2^ Energy and Environmental Science and Technology Idaho National Laboratory Idaho Falls ID 83415 USA

**Keywords:** ammonia synthesis, CO_2_ reduction, natural gas upgrading, protonic ceramic electrochemical fuel cells, sustainable chemical synthesis

## Abstract

Protonic ceramic electrochemical cells (PCECs) have been intensively studied as the technology that can be employed for power generation, energy storage, and sustainable chemical synthesis. Recently, there have been substantial advances in electrolyte and electrode materials for improving the performance of protonic ceramic fuel cells and protonic ceramic electrolyzers. However, the electrocatalytic materials development for synthesizing chemicals in PCECs has gained less attention, and there is a lack of systematic and fundamental understanding of the PCEC reactor design, reaction mechanisms, and electrode materials. This review comprehensively summarizes and critically evaluates the most up‐to‐date progress in employing PCECs to synthesize a wide range of chemicals, including ammonia, carbon monoxide, methane, light olefins, and aromatics. Factors that impact the conversion, selectivity, product yield, and energy efficiencies are discussed to provide new insights into designing electrochemical cells, developing electrode materials, and achieving economically viable chemical synthesis. The primary challenges associated with producing chemicals in PCECs are highlighted. Approaches to tackle these challenges are then offered, with a particular focus on deliberately designing electrode materials, aiming to achieve practically valuable product yield and energy efficiency. Finally, perspectives on the future development of PCECs for synthesizing sustainable chemicals are provided.

## Graphical abstract

Protonic ceramic electrochemical cells (PCECs) enable the reduction of nitrogen to ammonia, the reduction of carbon dioxide to value‐added carbon‐containing chemicals, and the dehydrogenation of alkanes to high‐value olefins and aromatics. In this review, the progress in employing PCECs for synthesizing chemicals is discussed. Additionally, current challenges and future perspectives are presented to achieve high‐efficiency chemical synthesis in PCECs.

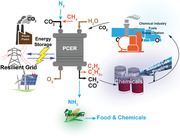

## 1 Introduction

Electrochemical cells using proton‐conducting ceramic electrolyte membranes have recently been revived, which is attributed to the numerous studies that have demonstrated that proton‐conducting ceramic fuel cells and electrolysis cells can be readily manufactured, and their performances have been significantly enhanced in the last decade.^[^ ^1^, ^2^, ^3^, ^4^, ^5^, ^6^, ^7^, ^8^, ^9^, ^10^, ^11^, ^12^, ^13^ ^]^ Protonic ceramic electrochemical cells (PCECs) are also emerging for synthesizing chemicals more efficiently and sustainably.^[^ ^14^, ^15^, ^16^, ^17^, ^18^, ^19^, ^20^, ^21^, ^22^, ^23^, ^24^, ^25^ ^]^ PCECs are versatile and capable of synthesizing broad and diversified chemicals from earth‐abundant feedstocks (e.g., H_2_O and N_2_) and CO_2_, and upgrading natural gas (methane) with reduced carbon footprint and enhanced energy efficiency. PCECs offer various fascinating advantages that distinguish them from other chemical production technologies. These advantages include the intermediate operating temperature (300–600 °C) that thermodynamically and kinetically favors the electrode chemistry while potentially allowing the co‐utilization of renewable power and waste heat.^[^ ^26^ ^]^ Therefore, PCECs can be readily integrated with renewable or nuclear power plants to convert electrical energy. PCECs could also be integrated with fossil assets to capture and utilize CO_2_ and waste heat. Moreover, the intermediate operating temperature will relax the system design and control,^[^ ^27^ ^]^ consequently reducing both capital and operational costs. The modular configuration of PCECs enables them to be deployed as distributed systems, which further facilitates their integration with renewable power plants and enables the use of various feedstocks, creating substantial market potential for renewable energy sectors and chemical industries.

**Figure 1:**
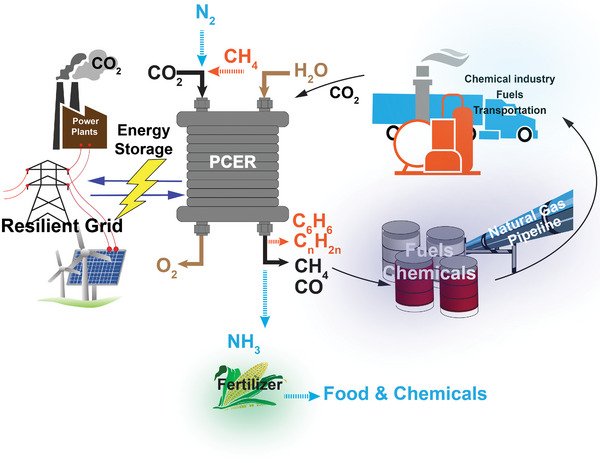
Applications of PCECs for sustainable chemical synthesis.

As schematically illustrated in **Figure** **1** and summarized in **Table** **1**, PCECs have been adapted to various applications, including synthesizing ammonia that can be utilized as fertilizers to improve crop yield, converting CO_2_ to sustainable CO, CH_4_, and syngas, and upgrading CH_4_ to high‐value aromatics and light olefins. The reactor configurations for these three applications are displayed in Table 1. Additionally, Table 1 presents the reactions that occur at both positive electrodes and negative electrodes. Table 1 also summarizes the representative electrode materials for these reactions and the state‐of‐the‐art performances. As shown in Figure 1, the PCEC can function as a fuel cell in its reversed mode to interconvert chemical energy and electrical energy; thus, the PCEC can be employed for the chemical energy storage system. Numerous lab‐scale PCECs have been demonstrated to realize these applications.^[^ ^5^, ^14^, ^17^, ^23^, ^28^ ^]^ These previous studies have centered on proving the concept of producing chemicals in PCECs. However, the reaction thermodynamics, energy efficiency, potential reaction mechanisms, critical barriers, and potential strategies to overcome the challenges are not discussed in detail. For example, the energy efficiency of synthesizing ammonia in PCECs has not been determined and compared with the conventional Haber–Bosch (HB) process. The electrochemical reactions in PCECs have not been clearly and systematically elaborated. Additionally, as listed in Table 1, despite synthesizing chemicals in PCECs displays multiple compelling benefits, challenges concurrently exhibit, some of which have been noted while some issues have not been recognized. Therefore, comprehensively summarizing these challenges and disclosing the corresponding strategies can unlock additional opportunities for synthesizing chemicals in PCECs.

**Table 1:** Summary of PCECs for synthesizing chemicals

	Ammonia Synthesis	CO_2_ reduction	Natural gas conversion
Configuration	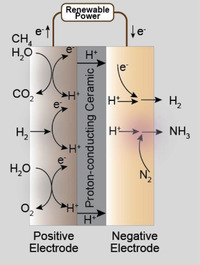	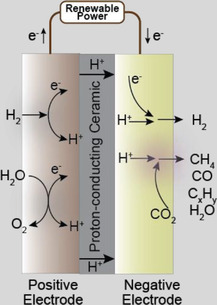	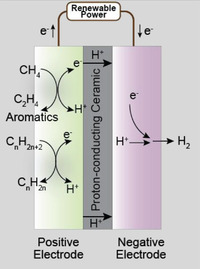
Reactions at the positive electrode and representative positive electrode materials	Steam methane reforming and water gas shift reaction: BaZr_0.8_Y_0.2_O_3+*δ*_+Ni^[^ ^1^ ^]^ H_2_ oxidation reaction: BaZr_0.8_Y_0.2_O_3+*δ*_+Ni or BaCe_0.7_Zr_0.1_Y_0.1_Yb_0.1_O_3+*δ*_+Ni^[^ ^1^, ^2^, ^29^ ^]^ Oxygen evolution reaction: BaCo_0.4_Fe_0.4_Zr_0.1_Y_0.1_O_3+*δ*_^[^ ^22^ ^]^ PrBa_0.5_Sr_0.5_Co_1.5_Fe_0.5_O_5+*δ*_^[^ ^7^ ^]^ Ba_1−x_Gd_0.8_La_0.2+x_Co_2_O_6−*δ*_^[^ ^30^ ^]^	H_2_ oxidation reaction: BaZr_0.8_Y_0.2_O_3+*δ*_+Ni or BaCe_0.7_Zr_0.1_Y_0.1_Yb_0.1_O_3+*δ*_+Ni^[^ ^1^, ^2^, ^29^ ^]^ Oxygen evolution reaction: BaCo_0.4_Fe_0.4_Zr_0.1_Y_0.1_O_3+*δ*_^[^ ^22^ ^]^ PrBa_0.5_Sr_0.5_Co_1.5_Fe_0.5_O_5+*δ*_^[^ ^7^ ^]^ Ba_1−x_Gd_0.8_La_0.2+x_Co_2_O_6−*δ*_^[^ ^30^ ^]^	Methane dehydroaromatization: Mo/H‐MCM‐22^[^ ^23^ ^]^ Alkane dehydrogenation: PrBa_0.5_Sr_0.5_Co_1.5_Fe_0.5_O_5+*δ*_^[^ ^7^ ^]^
Reactions at the negative electrode and representative negative electrode materials	Nitrogen reduction reaction: VN‐Fe^[^ ^14^ ^]^ Hydrogen evolution reaction (HER, side reaction)	CO_2_ reduction reaction: PBM—BZY/Ir—O or PBM—BZY/Ir—Ir^[^ ^4^ ^]^ HER (side reaction)	HER BaZr_0.8_Y_0.2_O_3+*δ*_+Ni or BaCe_0.7_Zr_0.1_Y_0.1_Yb_0.1_O_3+*δ*_+Ni^[^ ^1^, ^2^, ^29^ ^]^
Advantages	Intermediate operating temperatures (400–500 °C) thermodynamically and kinetically favor NH_3_ synthesis	High CO_2_ conversion High chemical production rate Low overpotentials	Enhanced methane conversion Co‐generation of hydrogen Lower operating temperature
Disadvantages	HER is thermodynamically more favorable than NRR High temperature favors NH_3_ decomposition	High temperature favors thermochemical CO_2_ reduction High temperature favors HER	Potentially exacerbated coking Complicated catalyst integration
State of the art	>5 × 10^−9^ mol cm^−2^ s^−1[^ ^31^ ^]^	>95% selectivity to CO/CH_4_ at 400 °C^[^ ^28^ ^]^	CH_4_ conversion > 11% >40 h of stable operation at 710 °C^[^ ^23^ ^]^
Critical Barriers	Low NH_3_ Faradaic selectivity; Severe HER	Severe HER; Poor stability; Low Faradaic selectivity toward C2 and C2+ products	Low methane conversion; Coking

Herein, to provide insights into designing PCEC reactors, probing reaction mechanisms, identifying associated challenges, and proposing corresponding strategies, the most up‐to‐date PCECs are comprehensively analyzed. Depending on the chemicals that can be produced in PCECs, the PCECs are divided into three main categories, including nitrogen reduction in PCECs for ammonia synthesis, CO_2_ reduction reaction for producing carbon‐containing chemicals, and PCECs for upgrading natural gas (methane and other alkanes) to aromatics and light olefins. This review aims to present the PCEC design and configurations that have been developed and experimentally validated for these three applications. The corresponding reactions thermodynamics and mechanisms will be discussed. The recent progress and achievements of synthesizing chemicals in PCECs are summarized. We will subsequently identify and discuss the factors that impact the conversion, product yield, energy efficiency, and durability, guiding the future research and development of PCECs. Additionally, the challenges associated with chemical synthesis in PCECs will be outlined. Finally, the review offers strategies to overcome these challenges concerning materials design and synthesis, PCEC reactor design, and operating conditions.

## 2 Nitrogen Reduction Reaction for Ammonia Synthesis

There is significant commercial and economic incentive to produce ammonia more efficiently and sustainably, as ammonia is widely used as fertilizer, refrigerant gas, the building block for other chemicals, and fuel for power generation.^[^ ^32^, ^33^, ^34^ ^]^ The ammonia production industry based on the conventional Haber–Bosch (HB) process is responsible for >1.0% global greenhouse gas (GHG) emissions,^[^ ^32^ ^]^ striking the development of alternative ammonia synthesis technologies and the optimization of conventional HB ammonia synthesis processes. The main challenges of the HB process arise from 1) harsh operating conditions, especially high pressure (e.g., 300 bar), which is required to activate the N_2_ triple bond and thermodynamically favor the ammonia production,^[^ ^35^, ^36^ ^]^ and 2) hydrogen is produced primarily from natural gas via steam reforming,^[^ ^37^, ^38^, ^39^ ^]^ which is of high emissions and energy‐intensive. PCECs with different configurations have been employed to address these challenges by integrating electrochemical ammonia synthesis with other thermochemical and electrochemical processes, aiming to increase ammonia yield while simultaneously enhancing overall energy efficiency and reducing GHG emissions.

### 2.1 Configurations of PCECs for Ammonia Synthesis

**Figure 2:**
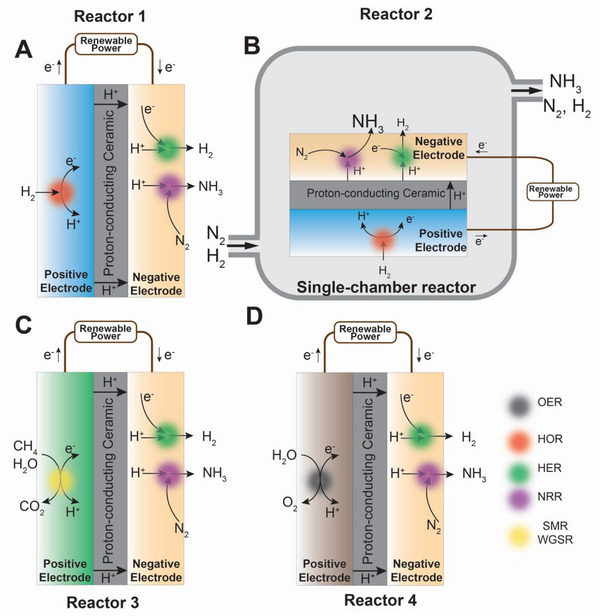
Schematic illustration of four primary PCEC configurations developed for ammonia synthesis. A) Reactor 1 that converts H_2_ and N_2_ to ammonia in a two‐chamber configuration, B) single‐chamber Reactor 2 that electrochemically promotes ammonia synthesis, C) Reactor 3 that intensifies steam methane reforming with ammonia synthesis, and D) Reactor 4 that converts H_2_O and N_2_ to ammonia. OER: oxygen evolution reaction. HOR: hydrogen oxidation reaction. HER: hydrogen evolution reaction. NRR: nitrogen reduction reaction. SMR: steam methane reforming. WGSR: water gas shift reaction.

Four major PCEC reactor configurations have been designed and validated for ammonia synthesis, which are schematically illustrated in **Figure** **2**. Some of these reactors integrate electrochemical/thermochemical processes and separation processes with ammonia synthesis, aiming to circumvent the thermodynamic limitations of ammonia synthesis that are typically encountered in a classical HB reactor. This integration also allows using renewable power for ammonia synthesis, which enhances the sustainability of ammonia production and reduces the carbon footprint of ammonia manufacturing industries. Ammonia produced via this sustainable approach can also serve as energy storage media or hydrogen carrier, which is attributed to its high energy density, high compressibility, carbon‐neutral nature, and lower transportation costs than hydrogen.^[^ ^40^ ^]^ When additional electricity is needed to supplement the intermittent renewable power sources, PCECs can function as fuel cells to directly convert ammonia to electrical energy without any changes to the configuration of PCECs.^[^ ^5^, ^17^ ^]^

Figure 2 shows that PCECs for synthesizing ammonia can be classified according to the configurations and feedstocks. Reactor 1 and Reactor 2 have been developed to electrochemically reduce nitrogen to ammonia to enhance the ammonia production rate under mild conditions, such as ambient pressure. Reactor 3 intensifies steam methane reforming with ammonia synthesis, eliminating the external reformer and separating the H_2_ stream from the CO_2_ stream, which consequently simplifies CO_2_ capture and reduces carbon footprint. Reactor 4 can convert H_2_O and N_2_ to ammonia via nitrogen reduction reaction and water electrolysis that provides alternative hydrogen.

The detailed processes, unique features, innovations, and challenges of these four reactors are outlined as follows:

In Reactor 1, H_2_ is fed to the positive electrode while N_2_ is delivered to the negative electrode. Under an electrical potential, protons transport to the negative electrode where N_2_ is reduced to ammonia. The chemisorption of both nitrogen and hydrogen on catalysts, and the binding energy of nitrogen with the catalyst, are essential to high ammonia yield. A high hydrogen coverage can poison the catalysts, while a lower hydrogen coverage is insufficient for nitrogen reduction.^[^ ^41^, ^42^ ^]^ Therefore, the polarization current density applied on PCECs, which determines the proton flux, can affect hydrogen coverage on the negative electrode and ammonia production rate. Additionally, as the negative electrode can have proton conduction, the hydrogen is primarily absorbed on the surface of proton conductors, which creates additional active sites on the metallic phase for the dissociative adsorption of nitrogen.^[^ ^43^ ^]^ Reactor 1 can therefore accelerate the kinetics of nitrogen reduction ammonia production. Reactor 1 can directly convert the electrical energy to chemical energy, which could circumvent the thermodynamic limits encountered in conventional HB reactors, eliminating the requirements of high‐pressure operation.^[^ ^44^, ^45^ ^]^ Therefore, the objective of employing Reactor 1 is to enhance ammonia yield under mild conditions (e.g., ambient pressure). However, Reactor 1 cannot address the issue associated with hydrogen production, which requires integrating Reactor 1 with hydrogen production units, including steam reforming of fossil fuels or renewable hydrogen production technologies (e.g., electrolysis, biomass, photoelectrochemical catalysis, and solar thermochemical H_2_).

Reactor 2 presents PCECs with a single‐chamber configuration that is similar to the conventional HB reactor counterpart, which can convert H_2_ and N_2_ to ammonia in a single chamber. The Reactor 2 configuration is also similar to the single‐chamber solid oxide fuel cells (SOFCs),^[^ ^46^, ^47^ ^]^ where a mixture of fuel and oxygen is supplied to the same chamber. The anode selectively oxidizes the fuel while the cathode selectively reduces O_2_, enabling the generation of power. The concept of designing electrodes with selective activity to enable power generation can assist in designing positive and negative electrodes of PCECs with a Reactor 2 configuration. A mixture of H_2_ and N_2_ is used as the feedstock in Reactor 2. The positive electrode can selectively oxidize H_2_ while the negative electrode can selectively reduce N_2_. Compared to the conventional HB process, the main difference is that an ammonia synthesis catalyst is applied as the negative electrode of PCECs. Applying a polarization current can control the hydrogen coverage on the catalyst and the binding energy of nitrogen, which consequently leads to a non‐Faradaic effect and significantly improve the ammonia yield.^[^ ^48^ ^]^ In terms of the hydrogen source, Reactor 2 displays the same characteristic as Reactor 1. Moreover, the ammonia synthesis in Reactor 2 is constrained by thermodynamics, which exhibits the same issue as the HB process. Therefore, the ammonia yield in Reactor 2 under ambient pressure is poor. It has been demonstrated Reactor 2 can operate at a slightly high operating pressure (e.g., 30 bar), allowing to significantly enhance ammonia production by applying a current.^[^ ^48^ ^]^ The central benefit of implementing Reactor 2 is to reduce the operating pressure of the HB process while achieving a higher ammonia yield.

Reactor 3 intensifies steam methane reforming with ammonia synthesis. Renewable power is utilized to pump the hydrogen from the positive electrode to the negative electrode, which shifts the methane reforming equilibrium toward a higher methane conversion.^[^ ^18^ ^]^ Under a specific current density, methane could achieve complete conversion at an intermediate temperature (e.g., 400–550 °C). The outlet gas stream will be CO_2_‐rich gas that can be significantly captured. This process intensification gives rise to multiple benefits. First, it eliminates the external reformer and simplifies the system. Additionally, this integrated reactor couples the endothermic reforming reactions with exothermic processes (e.g., nitrogen reduction, heat generated via Ohmic loss), creating a thermoneutral regime and dramatically enhancing the overall energy efficiencies. Moreover, this reactor separates the CO_2_ gas stream from the ammonia gas stream. The CO_2_ capture will reduce the emissions during ammonia production. Additionally, as CH_4_ is fed to the positive electrode along with CO_2_ produced, a highly active and CO_2_‐tolerant electrode material is required to achieve both high performance and durable operation. Therefore, rationally designed positive electrode for steam methane reforming is also crucial to achieving high cell performance and long‐term durability

Reactor 4 represents the ultimate alternative that could transform the energy‐intensive and high‐carbon emission HB process toward sustainable ammonia production. Ammonia can be directly produced from H_2_O and N_2_, which are cheap and abundant feedstocks, by using PCECs powered by renewable power sources. Oxygen evolution reaction occurs at the positive electrode, which has been well‐established for renewable hydrogen production.^[^ ^4^, ^5^, ^7^, ^30^ ^]^ Protons will then transport across the electrolyte membrane and react with nitrogen at the negative electrode, producing ammonia.

These four PCEC configurations primarily differ in the feedstocks fed to the positive electrode and corresponding reactions. All these reactors, which are powered by renewable electricity, can enhance the ammonia production rate by modulating hydrogen coverage on the catalyst and promoting the dissociative adsorption of nitrogen. Moreover, Reactor 1, Reactor 3, and Reactor 4 potentially bypass the thermodynamic constraints of ammonia synthesis in conventional HB reactors, allowing them to achieve a higher ammonia yield under mild conditions. Both Reactor 3 and Reactor 4 concurrently increase ammonia yield and reduce carbon emissions.

The proton‐conducting electrolyte membrane has minor electronic leakage under an oxidizing atmosphere, which might lead to low Faradaic efficiency and reduced ammonia production rate. Prior work on PCECs has identified that electronic leakage is attributed to the oxidation of electrolytes.^[^ ^5^, ^49^ ^]^ As both positive electrodes and negative electrodes of Reactors 1–3 are under a reducing atmosphere that has a much lower oxygen partial pressure, the electronic leakage can be significantly suppressed. The electronic charge carrier transference numbers of two typical electrolyte materials, BaZr_0.8_Y_0.2_O_3‐*δ*_ and BaCe_0.7_Zr_0.1_Y_0.1_Yb_0.1_O_3‐*δ*_, under a reducing atmosphere is lower than 0.01%, indicating the electronic leakage can be negligible. Therefore, both BaZr_0.8_Y_0.2_O_3‐*δ*_ and BaCe_0.7_Zr_0.1_Y_0.1_Yb_0.1_O_3‐*δ*_ can be employed as the electrolyte for Reactors 1–3. Considering BaZr_0.8_Y_0.2_O_3‐*δ*_ exhibits improved CO_2_ and H_2_O tolerance than BaCe_0.7_Zr_0.1_Y_0.1_Yb_0.1_O_3‐*δ*_, BaZr_0.8_Y_0.2_O_3‐*δ*_‐based PCECs can achieve better stability than that of BaCe_0.7_Zr_0.1_Y_0.1_Yb_0.1_O_3‐*δ*_‐based PCECs.

In Reactor 4, the positive electrode is exposed to an oxidizing atmosphere. The oxidization of electrolyte membrane can result in p‐type electronic conduction. Under the humidified oxidizing atmosphere (e.g., 10% H_2_O balanced with air), BaZr_0.8_Y_0.2_O_3‐*δ*_ exhibits an electronic charge carrier transference number of >30% and BaCe_0.7_Zr_0.1_Y_0.1_Yb_0.1_O_3‐*δ*_ has an electronic transference number of <15%,^[^ ^5^ ^]^ suggesting BaCe_0.7_Zr_0.1_Y_0.1_Yb_0.1_O_3‐*δ*_ is a better electrolyte membrane for Reactor 4. Additionally, reducing oxygen partial pressure, increasing steam partial pressure, and decreasing operating temperature favor electrolyte hydration and inhibit oxidization, which will reduce electronic leakage.

### 2.2 Thermodynamics of Ammonia Synthesis in PCECs

**Figure 3:**
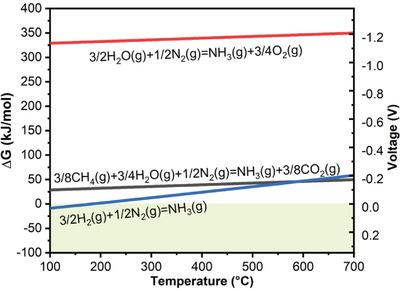
Thermodynamics and corresponding reversible electrochemical potential of three ammonia synthesis reactions calculated under the standard pressure.

**Figure** **3** shows the thermodynamics and reversible electrochemical potential required to electrochemically reduce nitrogen to ammonia. The Gibbs free energy change (Δ*G*) represents the minimum electrical energy consumption. The voltage denotes the minimum applied voltage (onset voltage) to drive the ammonia synthesis. Ammonia can be produced if the applied voltage is more negative (i.e., more cathodic) than that shown in Figure 3. The negative voltage indicates the reactions shown in Figure 3 cannot occur spontaneously. Electrical energy is consumed to break the associated chemicals for ammonia synthesis.

At an operating temperature of <180 °C, PCECs with H_2_ and N_2_ as the feedstocks possess a positive onset voltage, suggesting PCECs could potentially co‐generate electricity and ammonia. However, due to the sluggish kinetics of nitrogen reduction, especially at such a low operating temperature, the co‐generation of power and ammonia is not facile. At a typical PCEC operating temperature of 300–500 °C, converting H_2_ and N_2_ to ammonia consumes the least electrical energy. Converting CH_4_, H_2_O, and N_2_ to ammonia requires a slightly higher amount of electrical energy, which is attributed to the highly endothermic nature of the steam methane reforming. The total energy consumption of ammonia synthesis via the HB process is ≈500 kJ mol^−1^,^[^ ^32^ ^]^ which is much higher than the electrical energy consumption of Reactors 1–3 that is projected based on thermodynamics. As extensive electrical energy is utilized for water electrolysis, the energy consumption of Reactor 4 is much higher than Reactors 1–3, which is similar to the HB process. Therefore, A higher voltage (>1.1 V) is applied to drive the ammonia production in Reactor 4. Despite Reactor 4 consuming much higher electrical energy than the other reactors, ammonia produced in Reactor 4 is carbon‐free and sustainable. With the broad deployment of renewable power plants, the renewable electricity cost has been drastically reduced, which stimulates sustainable ammonia production by converting H_2_O and N_2_.

### 2.3 Current Progress in Utilizing PCECs for Ammonia Synthesis and Associated Challenges

PCECs with all these four configurations have been experimentally validated for ammonia synthesis. **Table** **2** presents a summary of notable PCECs for ammonia synthesis. An ammonia production rate of >5.0 × 10^−9^ mol cm^−2^ s^−1^ has been demonstrated in Reactor 1,^[^ ^31^ ^]^ which is higher than that of Reactor 3 and Reactor 4. At a relatively high operating pressure (e.g., 50 atm), the PCEC with the Reactor 2 configuration achieves an ammonia production rate of ≈ 5 × 10^−9^ mol cm^−2^ s^−1^,^[^ ^48^ ^]^ which is similar to the performance realized in Reactor 1. However, under ambient operating pressure, Reactor 2 does not achieve a higher ammonia production rate than that achieved in Reactor 1. Additionally, PCECs with the Reactor 4 configuration obtain lower ammonia production rates than other PCECs, which could be ascribed to the electronic leakage of electrolytes. The ammonia synthesis was conducted under a relatively low current density (e.g., <100 mA cm^−2^). Prior work on PCECs has recognized that the electronic leakage tends to be more severe at a current density of <100 mA cm^−2^ as the p‐type electronic conduction of the electrolyte creates a H_2_ permeation flux from the negative electrode to the positive electrode, leading to high electronic leakage and low ammonia production rate.^[^ ^5^, ^7^ ^]^

**Table 2:** A summary of notable PCECs with four configurations for ammonia synthesis

	N_2_ reduction electrode (Negative electrode)	Counter electrode (Positive Electrode)	Electrolyte	Atmosphere	NH_3_ production rate [mol cm^−2^ s^−1^] x 10^9^	NH_3_ Faradaic Efficiency [%]	Ref.
Reactor1	Ba_0.5_Sr_0.5_Co_0.8_Fe_0.2_O_3−*δ*_	NiO—BaCe_0.85_Y_0.15_O_3‐*δ*_	BaCe_0.85_Y_0.15_O_3‐*δ*_	N_2_|H_2_	4.10	N/A	[50]
	Cu film	NiO‐CeO‐ BaZr_0.7_Ce_0.2_Y_0.1_O_2.9_	BaZr_0.7_Ce_0.2_Y_0.1_O_2.9_	N_2_|H_2_	1.71	<2.7	[51]
	Pt—Fe oxide‐ BaCe_0.5_Zr_0.3_Y_0.16_Zn_0.04_O_3‐*δ*_	NiO‐ BaCe_0.5_Zr_0.3_Y_0.16_Zn_0.04_O_3‐*δ*_	BaCe_0.5_Zr_0.3_Y_0.16_Zn_0.04_O_3‐*δ*_	N_2_|H_2_	3.95	N/A	[52]
	Pd	Pd	SrCe_0.95_Yb_0.05_O_3_	N_2_|H_2_	4.50	N/A	[53]
	Ag—Pd	Ag—Pd	Ba_3_Ca_0.9_Nd_0.28_Nb_1.82_O_9‐*δ*_	N_2_|H_2_	2.16	N/A	[54]
	Ag—Pd	Ag—Pd	BaCe_0.8_Gd_0.1_Sm_0.1_O_3‐*δ*_	N_2_|H_2_	5.82	N/A	[31]
Reactor 2	The BASF S6‐10RED	Ag	CaIn_0.1_Zr_0.9_O_3‐*δ*_	N_2_|H_2_	5.0	>100% (Non‐Faradaic effect)	[48]
	Rh film	NiO‐ BaCe_0.2_Zr_0.7_Y_0.1_O_3‐*δ*_	BaCe_0.2_Zr_0.7_Y_0.1_O_3‐*δ*_	Wet N_2_|H_2_	2.89	N/A	[55]
Reactor 3	VN‐Fe	Ni‐ BaCe_0.2_Zr_0.7_Y_0.1_O_3‐*δ*_	BaZr_0.8_Ce_0.1_Y_0.1_O_3‐*δ*_	N_2_|CH_4_, H_2_O	1.88	5.32	[14]
Reactor 4	Ru‐Ag/MgO	Pd	SrCe_0.95_Yb_0.05_O_3−*δ*_	N_2_, He|H_2_O, He	0.0003	N/A	[56]
	La_0.6_Sr_0.4_Co_0.2_Fe_0.8_O_3‐*δ*_	La_0.6_Sr_0.4_Co_0.2_Fe_0.8_O_3_‐ _*δ*_	BaZr_0.8_Y_0.2_O_3‐*δ*_	N_2_, He|H_2_O, Ar	0.085	0.33	[57]

**Figure 4:**
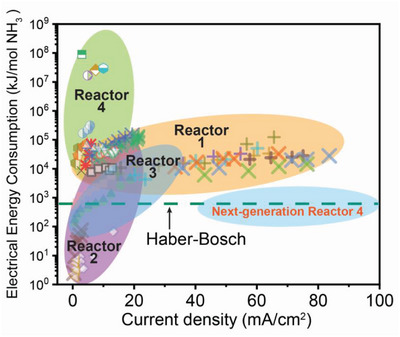
Electrical energy consumption of synthesizing ammonia in PCECs.^[^ ^14^, ^48^, ^50^, ^51^, ^52^, ^55^, ^56^, ^59^ ^]^ The electrical energy consumption is determined based on the experimental results reported in papers published in the last two decades. Cross and star symbols represent the PCECs with Reaction 1 configuration. Open symbols represent single‐chamber PCECs with Reactor 2 configuration. Solid symbols denote PCECs with Reactor 3 configuration. Half‐solid and half‐open symbols stand for PCECs with Reactor 4 configuration. Next‐generation Reactor 4 indicates the targets of synthesizing ammonia in PCECs. The green dash line represents the total energy consumption for the Haber–Bosch process.

To better understand the current status and evaluate the economic viability of synthesizing ammonia in PCECs, determine the potential challenges, and identify the approaches to addressing them, the guiding performance metric for comprehensively evaluating PCECs should be established. This metric should synergize multiple performance indicators, including the current density, voltage, Faradaic selectivity, and ammonia production rate. Prior efforts have focused on achieving a high production rate or enhancing Faradaic selectivity toward ammonia, which cannot completely represent the practical value of producing ammonia in PCECs. Here, we perform an analysis of the electrical energy consumed for producing one mole of ammonia that can be the guiding performance metric, which provides some insights that may inform future PCEC development. This metric has been used for evaluating the electrical energy consumption of other electrochemical ammonia synthesis technologies.^[^ ^58^ ^]^ Therefore, **Figure** **4** is presented to report the electrical energy consumption of synthesizing ammonia in PCECs, which comprehensively summarizes the performances of PCECs with the consideration of current density, selectivity toward ammonia, applied voltage, and ammonia production rate. The electrical energy consumption of ammonia synthesis is determined by using the following equation.

(1) $$\begin{array}{c} \begin{array}{c} \mathrm{Electrical}\;\mathrm{energy}\;\mathrm{consumption}\;\left(\mathrm{kJ}/{\mathrm{mol}}_{{\mathrm{NH}}_{3}}\right)=\frac{j\;\left(\frac{\mathrm{mA}}{{\mathrm{cm}}^{2}}\right)\times V\left(V\right)}{r_{{\mathrm{NH}}_{3}}\left(\frac{\mathrm{mol}}{s\cdot {\mathrm{cm}}^{2}}\right)\times 10^{6}} \end{array} \end{array}$$

Figure 4 presents our survey of the published work that simultaneously reports the applied voltage, current density, and ammonia production rate.^[^ ^14^, ^48^, ^50^, ^51^, ^52^, ^55^, ^56^, ^59^ ^]^ Some published papers did not report either the applied voltage or the current density, which cannot be used to thoroughly evaluate its performances,^[^ ^16^, ^31^, ^54^, ^60^, ^61^, ^62^, ^63^, ^64^, ^65^, ^66^ ^]^ especially the electrical energy consumption. At a current density of <10 mA cm^−2^, PCECs with a configuration of Reactor 3 and Reactor 2 have led to electrical energy consumption that is lower than the conventional HB process.^[^ ^14^, ^55^, ^59^ ^]^ Once the current density is increased to 20 mA cm^−2^, its electrical energy consumption is ten times as high as that at a current density of 10 mA cm^−2^, which is due to the decreased ammonia Faradaic selectivity. Increasing the current density can increase the overpotential attributed to the negative electrode. The more cathodic potential at the negative electrode can facilitate the HER and consequently reduce the Faradaic selectivity toward ammonia. PCECs with a configuration of Reactor 1 are intensively studied for ammonia synthesis as this PCEC architecture simplifies the reactor design and operation.^[^ ^52^ ^]^ Reactor 1 also delivers the highest current density. However, its electrical energy consumption is more than ten times higher than that of the HB process, mainly ascribed to the low Faradaic selectivity toward ammonia at a relatively high current density. It is moreover clear that the PCECs with a configuration of Reactor 4 consume a much higher amount of electrical energy than the other configuration as the applied voltage for water electrolysis alone is dramatically high, which is consistent with the thermodynamics shown in Figure 3. Additionally, the current density of Reactor 4 is much lower than other reactors. Despite Reactor 4 requiring much higher energy consumption, Reactor 4 does not need any fossil fuel as the feedstock and eliminates CO_2_ emissions. The electrical energy consumption in Reactor 4 could be dramatically reduced once the HER is suppressed and ammonia selectivity is improved. As shown in Figure 4, it is expected that PCECs with a next‐generation Reactor 4 configuration and optimized electrode materials can produce ammonia at a higher current density and selectivity, leading to comparable or lower energy consumption than the HB process. The strategies to achieve Next‐generation Reactor 4 will be discussed in great detail in later sections of this review.

### 2.4 Ammonia Synthesis Mechanisms

**Figure 5:**
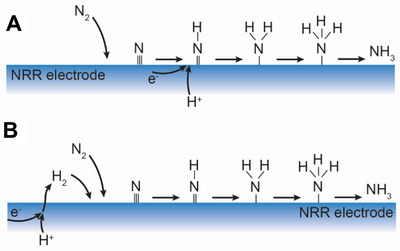
Potential NRR pathways in PCECs. A) Dissociative electrochemical mechanism (Pathway 1). B) Associate thermochemical mechanism (Pathway 2).

**Figure** **5** presents two primary nitrogen reduction and ammonia production pathways that occur at PCEC negative electrodes. The relatively high operating temperature (300–600 °C) of PCECs drives the N≡N bond cleavage; thus, the dissociative nitrogen reduction mechanism is relatively favorable. Furthermore, the NRR electrode is usually polycrystalline metals or metallic nanoparticles (e.g., Ru) with stepped surfaces, which favors nitrogen reduction via the dissociative mechanism.^[^ ^58^ ^]^ These two pathways presented in Figure 5 are based on the dissociative mechanism. However, these two pathways can lead to distinct ammonia selectivity. Pathway 1 (Figure 5A) shows the electrochemical ammonia synthesis via the dissociative mechanism. The proton could directly take electrons and reduce the dissociative adsorbed nitrogen on the catalyst to ammonia. The direct electrochemical ammonia synthesis does not require the stoichiometric amount of gaseous hydrogen production. Thus, this pathway can result in a much higher Faradaic selectivity. To favor Pathway 1, the NRR electrode should inhibit HER while exhibiting good electrocatalytic activity to reduce nitrogen. Pathway 2 displays the conventional HB reaction where nitrogen is reduced by the gaseous hydrogen produced via HER. Thermochemical catalytic ammonia synthesis dominates overall ammonia production. Therefore, the H_2_/N_2_ ratio is essential for the ammonia production rate as the overall yield will obey the thermodynamics of thermochemical ammonia synthesis. The PCECs will have to operate at a high current density to achieve a H_2_/N_2_ ratio that approaches 3/1 and thereby increasing ammonia yield. However, in these scenarios, the ammonia Faradaic selectivity is very poor as a significant amount of hydrogen cannot be utilized.

### 2.5 Challenges Associated with Ammonia Synthesis in PCECs

#### 2.5.1 Low Faradaic Selectivity toward Ammonia

PCECs have been demonstrated for ammonia synthesis over the last decades. The ammonia production rates typically range from 10^−10^ to 10^−8^ mol cm^−2^ s^−1^, which are higher than that obtained in low‐temperature (<100 °C) electrochemical devices.^[^ ^62^ ^]^ However, the Faradaic selectivity toward ammonia is low, which is ascribed to the parasitic HER. There is a lack of rationally designed negative electrodes for ammonia synthesis. As summarized in Table 2, perovskite‐based oxides or proton‐conducting oxide‐metal composite or metal, for instance, Ba_0.5_Sr_0.5_Co_0.8_Fe_0.2_O_3−*δ*_, Pd, Ag—Pd, and Rh, are directly applied as the negative electrodes.^[^ ^43^, ^48^, ^50^, ^55^, ^62^, ^66^ ^]^ These materials also favor HER, inevitably leading to significant hydrogen production and poor selectivity to ammonia. Furthermore, the current PCEC operating temperatures (e.g., 500–600 °C) are slightly high, which favors the dissociative adsorption of nitrogen. However, the decomposition of ammonia is also favorable at 500–600 °C, further reducing the selectivity toward ammonia. Therefore, both the negative electrode and operating temperatures can influent the HER and ammonia selectivity.

#### 2.5.2 Nitrogen Reduction Reaction Pathway

It has been noted that increasing the current density increases the ammonia production rate because increasing the current density can enhance the H_2_/N_2_ ratio and thereby could facilitate ammonia production via the HB process. On the other hand, increasing current density also affects the hydrogen and nitrogen coverage on the negative electrode, enhancing the electrochemical ammonia production rate. However, there is no well‐established tool to differentiate the ammonia produced via electrochemical nitrogen reduction from the HB process (i.e., two pathways shown in Figure 5). Therefore, it is challenging to directly prove electrochemical ammonia synthesis in PCECs.

#### 2.5.3 Discrepant Faradaic Selectivity

Additionally, discrepancies in the Faradaic selectivity toward ammonia still exist. An ammonia selectivity of >50% has been demonstrated in certain PCECs. In comparison, some studies present an ammonia selectivity of <1%,^[^ ^14^, ^48^, ^50^, ^51^, ^52^, ^55^, ^56^, ^59^ ^]^ suggesting it is necessary to develop more rigorous procedures to test the PCECs and measure the ammonia production rate, which will subsequently provide more reliable PCEC performances.

### 2.6 Strategies to Enhance Ammonia Selectivity and Yield, as well as Energy Efficiency

The intermediate operating temperatures of PCECs offer the thermodynamic and kinetic sweet spot for high‐efficiency ammonia synthesis. However, the catalysts that have been developed for the HB process cannot be directly applied to PCECs, which is due to the catalyst is not electronically conductive. The HB catalysts typically fall into two categories, including fused‐iron and supported metallic catalysts. Fused‐iron catalysts are derived from iron oxides, which are not highly conductive. The supported metallic catalysts are generally activated carbon or metal oxide‐supported ruthenium or cobalt.^[^ ^68^ ^]^ The activated carbon cannot be used for PCECs as the carbon can be easily oxidized during fabrication. Additionally, as the weight percentage of the metallic phase is normally <10% while the metal oxides are not electronically conductive, the metal‐oxide‐supported metallic catalyst cannot be applied on PCECs as the negative electrode. However, as PCECs can operate at temperatures similar to the HB process, the catalyst design and development strategies established for the HB process might be the starting points to develop negative electrode materials for PCECs, especially the most recent HB catalysts that are active under mild conditions.^[^ ^69^, ^70^ ^]^

The proton conductivity of the electrolyte is essential for providing sufficient protons/hydrogen to the negative electrode for reducing nitrogen. There has been extensive work on enhancing its intrinsic conductivity and fabricating thin electrolytes to minimize Ohmic losses.^[^ ^28^, ^63^ ^]^ A current density of >500 mA cm^−2^ can be easily achieved in protonic ceramic electrolysis cells with a low Ohmic loss.^[^ ^4^, ^7^, ^17^, ^21^, ^22^, ^30^ ^]^ Therefore, future work should focus on deliberately designing and synthesizing negative electrodes to improve the catalytic activity of negative electrodes and suppress the HER. The negative electrodes for PCECs should be electronically conductive to expedite charge transfer. Moreover, it should exhibit excellent catalytic activity to activate nitrogen and produce ammonia. It should also simultaneously hinder HER. Prior works have explored various metallic catalysts to achieve a higher ammonia yield while understating the impacts of catalyst support or the second phase of a composite electrode is limited.^[^ ^61^, ^64^ ^]^ Tailoring both the metallic phase and the support could diminish the active sites for HER while enhancing the active sites for nitrogen reduction.

**Figure 6:**
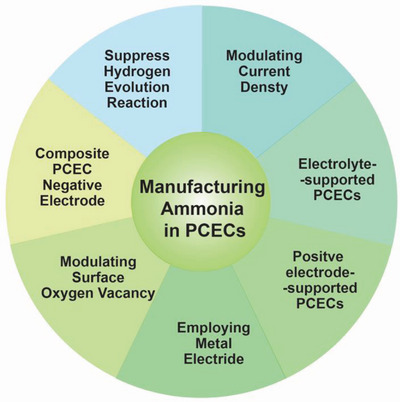
Summary of the strategies proposed for enhancing ammonia production in PCECs.

Herein, as shown in **Figure** **6**, this review intends to provide a set of strategies from different perspectives to enhance ammonia selectivity and yield. These strategies include deliberately designing NRR electrocatalysts and negative electrodes of PCECs, optimizing PCEC architectures, and modulating the PCEC operation conditions.

#### 2.6.1 NRR Electrocatalysts and PCEC Negative Electrode Design

Applying metal nitride as the NRR catalyst is expected to enhance ammonia selectivity and yield. The proton directly reacts with the lattice nitrogen to produce ammonia.^[^ ^65^, ^66^, ^67^, ^68^, ^69^ ^]^ Gaseous nitrogen will then adsorb on the metal nitride to replenish the lattice nitrogen. Experimental evidence, for instance, isotope‐based experiments, should be performed to prove that the gaseous nitrogen can replenish the nitrogen‐vacancy in transitional metal nitride.^[^ ^70^ ^]^ Furthermore, due to the low electronic conductivity of metal nitride, employing metal nitride might increase overpotentials and electrical energy consumption. To tackle this issue, metal nitride could be dispersed on a mixed electronic and protonic support to create electrochemically active sites. For example, transition metal‐doped BaCe_x_Zr_0.8‐x‐y_TM_y_Y_0.2_O_3_ (BCZTMY, TM = Mn, Ni, Fe) or SrCe_0.7_Zr_0.2_Eu_0.1_O_3‐δ_, which simultaneously displays electronic and protonic conduction, can be used as the electrode support.^[^ ^57^, ^71^ ^]^ The support should also be carefully engineered to inhibit HER.

Employing NRR catalyst with appropriately strong nitrogen binding with the catalysts to ensure high selectivity to ammonia and inhibit HER. According to the Brønsted–Evans–Polanyi relation, the dissociative adsorption of nitrogen requires strong binding energy on the surface of negative electrodes. However, enhancing the nitrogen binding cannot continually increase ammonia selectivity. The strong binding energy can lead to high desorption energy due to the scaling relations.^[^ ^72^, ^73^ ^]^ Furthermore, strengthening the nitrogen binding energy could lead to a higher overpotential and consequent low energy efficiency. It is therefore important to rigorously design the electrode with an optimal nitrogen binding to maximize ammonia production and energy efficiency. It is suggested to perform molecular modeling to determine the nitrogen binding energy, assisting in designing new catalysts.

It has been recognized that the Ru sites with oxygen vacancies can serve as the active sites that stabilize *NNH, destabilize *H, and enhance N_2_ adsorption, which suppresses HER and favors NRR.^[^ ^74^ ^]^ Therefore, oxygen vacancies on the surface of transition metal oxides could accelerate the electron charge transfer while it might not be active for HER; thus, increasing the oxygen vacancies of the oxide support can alter the NRR electrocatalytic activities.^[^ ^74^ ^]^ The oxides that can be applied as the support possess a high surface oxygen vacancy concentration and can also inhibit HER, implying they cannot transport protons as the proton conductor can facilitate HER.^[^ ^40^ ^]^ For example, the proton‐conducting oxides might not function well as the catalysts support as its high proton conductivity could lead to favorable HER. Therefore, ZrO_2_, CeO_2_, and acceptor‐doped ZrO_2_/CeO_2_ are expected to be effective catalyst support that can create active sites for nitrogen reduction to ammonia. Moreover, doping ZrO_2_ or CeO_2_ with an acceptor (e.g., Sm, Gd) can increase its oxygen vacancy concentration, which could improve its NRR activity.

#### 2.6.2 PCEC Architecture Design

##### Positive Electrode‐Supported PCECs

A positive electrode‐supported PCEC is expected to be an optimal architecture as this design offers substantial opportunities for using broad NRR electrocatalysts as the negative electrodes. The current PCECs mainly use Ni‐based cermet negative electrodes as the support. The researcher would try to use the Ni‐based cermet electrode as the NRR electrode, which favors HER and consequently leads to poor Faradaic selectivity toward ammonia. Alternatively, positive electrode‐supported PCECs can address the abovementioned challenges. A positive electrode scaffold with a thin electrolyte can be fabricated by conventional tape casting, dry pressing, or other coating technologies followed by sintering at a high temperature. The deliberately designed NRR electrocatalyst is directly coated on the electrolyte layer. This architecture allows the evaluation of various NRR electrocatalysts.

##### Electrolytes‐Supported PCECs

Alternatively, electrolyte‐supported PCECs offer the same advantage as that of positive electrode‐supported PCECs. As the electrolyte‐support PCECs normally use an electrolyte membrane with a thickness of 300–500 µm, which gives rise to 10 times higher ohmic resistance. Thanks to the recent advancements in highly‐conductive proton‐conducting oxides.^[^ ^9^, ^21^, ^29^ ^]^ an electrolyte with a thickness of 300–500 µm can achieve a current density of >500 mA cm^−2^. Thus, electrolyte‐supported architecture will not impact the ammonia production rate. However, electrical energy consumption will be slightly higher than that of positive electrode‐supported PCECs. The excess electrical energy consumption ascribed to ohmic loss is finally converted to heat, which can be utilized for water electrolysis and maintain the intermediate operating temperature. Therefore, the overall energy efficiency might not be drastically reduced. The electrolyte‐supported PCECs also simplify the preparation of positive electrodes. The conventional positive electrode can be easily coated on one side of the electrolyte, eliminating the complicated infiltration process. Therefore, electrolyte‐supported PCECs possess higher scalability than electrode‐supported PCECs.

#### 2.6.3 Operating Conditions

##### Current Density

**Figure 7:**
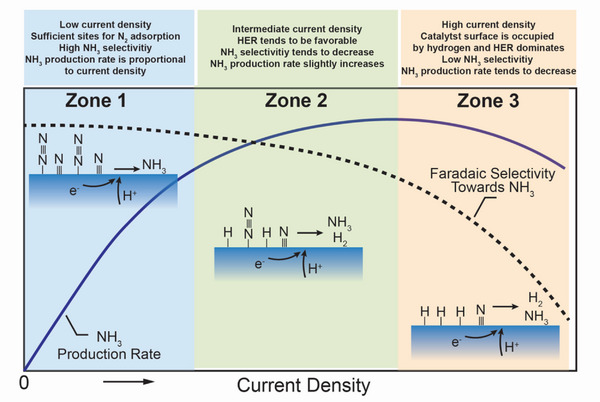
The ammonia production rate and Faradaic Selectivity toward ammonia as a function of the current density of PCECs.

As aforementioned, PCECs can facilely achieve a current density of >500 mA cm^−2^ for H_2_ production via steam electrolysis.^[^ ^4^, ^5^, ^7^ ^]^ However, increasing the current density cannot continuously increase the ammonia production rate.^[^ ^17^ ^]^ Therefore, it is essential to operate PCECs under an optimal current density to obtain high ammonia yield and energy efficiency. As shown in **Figure** **7**, at a relatively low current density, the negative electrode is covered with sufficient chemisorbed nitrogen that will be readily reduced by hydrogen/proton. Therefore, in Zone 1, increasing the current density can significantly enhance the ammonia production rate while a reasonable Faradaic selectivity toward ammonia is achieved. With further increasing the applied current density to Zone 2, the surface hydrogen concentration tends to increase and occupies the sites on the metal, enhancing the possibility of hydrogen production. The ammonia production rate can slightly increase due to the increased surface hydrogen concentration. However, as HER tends to be more facile, the Faradaic selectivity toward ammonia decreases. The ammonia production rate will peak at an intermediate current density, under which condition a maximum ammonia production rate could be obtained while the Faradaic selectivity and energy efficiency are not maximized. Therefore, the operating conditions that give rise to the highest ammonia yield might not be optimal for synthesizing ammonia. Zone 3 in Figure 7 displays the ammonia production rate and Faradaic selectivity at a higher current density. The catalyst surface is predominantly occupied by hydrogen, leading to severe hydrogen evolution, reduced ammonia production rate, and poor Faradaic selectivity toward ammonia.

##### H_2_ Gas Recycling

HER is parasitic and might be inevitable. Moreover, PCECs utilize intermittent renewable power to synthesize chemicals, suggesting the PCEC cannot always operate under the optimal condition to achieve the highest NH_3_ selectivity, which requires the PCECs have operational flexibility. Therefore, recycling the unreacted H_2_ and N_2_ to the negative electrode can be a feasible approach to enhancing NH_3_ yield and improving the utilization of protons for NH_3_ production. As shown in Figure 2, N_2_ is fed to the negative electrode; thus, the negative electrode outlet gas is a mixture of H_2_, N_2_, and NH_3_. NH_3_ can be captured via condensation and the unreacted H_2_ and N_2_ can be recycled back to PCECs. The H_2_ and N_2_ gas recycling can reduce the electric energy consumption of PCECs as a certain amount of H_2_ is recycled and PCECs can operate at a lower current density. Additionally, recycling unreacted H_2_ and N_2_ can adjust the H_2_‐to‐N_2_ ratio at the negative electrode, indicating the N_2_ feeding rate and the current density need to be dynamically adjusted to maximize NH_3_ production. Despite the H_2_ gas recycling has not been widely accepted, recycling H_2_ has been systematically analyzed for the HB reactor,^[^ ^84^, ^85^ ^]^ which concludes that the reactor can operate under a wide range of conditions by maintaining operational and ammonia production flexibilities.

## 3 CO_2_ Reduction Reaction in PCECs for Chemicals Production

### 3.1 CO_2_‐PCECs for Carbon‐Containing Chemical Production

Converting CO_2_ to value‐added chemicals via CO_2_ reduction reaction (CO2RR) simultaneously overcomes three critical energy and environmental challenges, including CO_2_ mitigation, sustainable chemical synthesis, and long‐term chemical energy storage. The wide deployment of CO_2_ reduction technologies reduces CO_2_ emissions while decreasing atmospheric CO_2_ concentration by producing sustainable chemical building blocks and curbing reliance on fossil fuels. Among various CO2RR technologies, electrochemical CO_2_ reduction reaction (ECO2RR) utilizes renewable electricity to power the electrochemical reactions, further reducing the carbon emissions ascribed to the power generation assets. Moreover, ECO2RR enables the interconversion between electrical energy and chemical energy, which can be employed for long‐term chemical energy storage.

PCECs have been demonstrated for the co‐conversion of CO_2_ and H_2_O into chemicals.^[^ ^20^, ^22^, ^31^, ^75^, ^76^, ^77^, ^78^, ^79^, ^80^ ^]^ Prior attempts to integrate CO_2_ reduction and H_2_O electrolysis have been plagued by the disparate temperatures between electrolyzers and CO_2_ reduction chemistries. Conventional solid oxide electrolyzers based on oxygen‐ion‐conducting membranes deliver exceptional CO_2_ conversion and efficiency but require operating temperatures above 700 °C at which the reaction of CO_2_ reduction to hydrocarbons (e.g., CH_4_) becomes thermodynamically unfavorable. Therefore, the end product will be limited to syngas (the mixture of H_2_ and CO).^[^ ^81^, ^82^, ^83^, ^84^ ^]^ Similarly, aqueous proton‐exchange membrane (PEM) CO_2_ electrolyzers and anion exchange membrane (AEM) CO_2_ electrolyzers can directly convert CO_2_ into high‐value chemicals at temperatures below 100 °C, but their CO_2_ reduction is kinetically limited.^[^ ^85^, ^86^, ^87^ ^]^ PCECs overcome these issues by providing high proton conductivity with doped ceramic membranes at versatile operating temperatures and conditions compatible with heterogeneous gas‐phase CO_2_ hydrogenation to hydrocarbons and CO_2_ conversion to CO. The high‐flux proton generated at an intermediate operating temperature (300–600 °C) from water electrolysis, the versatility to selectively synthesize CH_4_ or CO, cost‐effective and platinum group metal (PGM)‐free catalysts, and high energy conversion efficiency—all demonstrate that the PCEC is the “holy grail” for efficient CO_2_ conversion and value‐added chemicals production.^[^ ^5^, ^26^ ^]^ The major advantage is that the highly active protons transported across the electrolyte membrane, which operates at an intermediate temperature (300–500 °C), encounter a favorable kinetic and thermodynamic regime for CO_2_ reduction.

**Figure 8:**
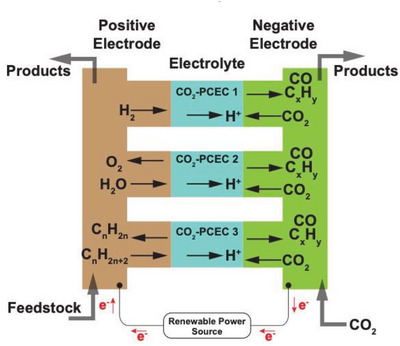
Schematic illustration of PCECs for carbon‐containing chemical synthesis via CO_2_ reduction reaction.

In the last decade, the advancements in proton‐conducting oxides have sparked the CO_2_ conversion and utilization in PCECs for synthesizing carbon‐containing chemicals. Various PCECs have been developed for reducing CO_2_ via hydrogen sourced from different feedstocks, including sustainable hydrogen, alkenes, and water.^[^ ^20^, ^22^, ^31^, ^75^, ^76^, ^77^, ^78^, ^79^ ^]^ As shown in **Figure** **8**, CO_2_ is fed to the negative electrode (CO_2_ reduction electrode) while the feedstocks for providing protons are delivered to the positive electrode. Like the PCECs designed for synthesizing ammonia, the reactions at the negative electrode will not vary with the reactants fed to the positive electrode, which simplifies the system design, enhances the feedstock flexibility, and relaxes the requirements for CO_2_ electroreduction catalysts. Furthermore, as shown in Figure 8, it is expected PCECs equipped with rationally designed CO_2_ electroreduction catalysts could be employed to produce carbon‐containing chemicals, including carbon monoxide, methane, and other hydrocarbons (C2 and C2+). However, converting CO_2_ to other hydrocarbons in PCECs has not been experimentally validated. Herein, we therefore center on discussing CO_2_ reduction to CO and CH_4_.

As shown in Figure 8, the PCECs for CO_2_ conversion can be categorized into three configurations according to the reactants delivered to the positive electrode, which include CO_2_‐PCEC 1, CO_2_‐PCEC 2, and CO_2_‐PCEC 3. CO_2_‐PCEC 1 aims to electrochemically reduce CO_2_ using hydrogen pumped from the positive electrode. The proton/hydrogen activates CO_2_ adsorbed on the negative electrode. The applied current density controls the surface hydrogen coverage and consequently affects the CO_2_ conversion and product yield. Additionally, the operating temperatures of PCECs range from 300 to 600 °C, which impacts the selectivity toward a particular product. The CO_2_ reduction to CO is endothermic; thus, CO is more prone to be produced at a relatively high operating temperature. CO_2_ reduction to methane is exothermic, which is therefore more thermodynamically favorable at low operating temperatures. However, the chemicals produced in PCECs are not solely determined by the operating temperatures. By modulating the kinetics of CO2RR over the negative electrode, CO can be favorable at relatively low operating temperatures while CH_4_ can also be selectively produced at slightly high operating temperatures.

CO_2_‐PCEC 2, as illustrated in Figure 8, displays the PCEC configuration for CO_2_ conversion with the highest sustainability, which enables the co‐conversion of CO_2_ and H_2_O into value‐added chemicals. Oxygen evolution reaction occurs at the positive electrode, while CO_2_ reduction occurs at the positive electrode. The chemicals produced via CO2RR will be ultimately oxidized to CO_2_ and H_2_O. Thus, synthesizing chemicals via co‐conversion of CO_2_ and H_2_O can achieve a net‐zero closed carbon cycle.

CO_2_‐PCEC 3 integrates the dehydrogenation of alkanes with CO_2_ reduction. Dehydrogenation of alkanes upgrades cheap and abundant hydrocarbons to value‐added light olefins. However, the conversion of alkanes is thermodynamically limited. CO_2_‐PCEC 3 pumps hydrogen from the positive electrode, shifting the dehydrogenation equilibrium toward higher conversion and enhancing the yield of light olefins. Furthermore, hydrogen/proton transports to the negative electrode where CO_2_ is reduced to value‐added chemicals. CO_2_ reduction to hydrocarbons is exothermic, which generates heat that could be directly used for the dehydrogenation of alkanes. Therefore, CO_2_‐PCEC 3 intensifies multiple reactions, which has multiple benefits, including improved product yield at both electrodes, enhanced energy efficiency, and raised CO_2_ conversion.

**Figure 9:**
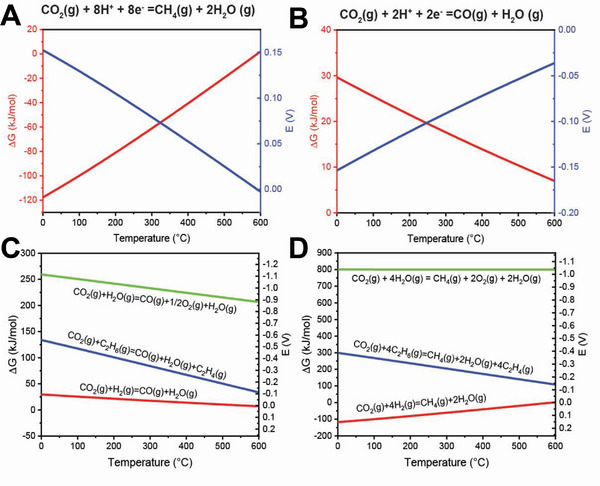
Thermodynamics of CO_2_ reduction in PCECs under standard atmosphere. A) The Gibbs free energy change of half‐cell reaction and corresponding CO_2_ reduction potentials versus the potential of HER (i.e., 2H^+^ + 2*e* ^−^ = H_2_(*g*)). The product of CO_2_ reduction is CH_4_. B) The Gibbs free energy change of half‐cell reaction and corresponding CO_2_ reduction potentials versus the potential of HER. The product of CO_2_ reduction is CO. The assumption in Figure 9A,B is that the potential of HER is zero and it does not change with operating temperatures. The equilibrium potential of CO_2_ reduction is calculated based on *E* = − Δ*G*/*nF*. C) The thermodynamics of CO_2_ reduction with various feedstocks and CO as the final product. D) The thermodynamics of CO_2_ reduction with various feedstocks and CH_4_ as the final product.

The thermodynamics of CO_2_ reduction to CH_4_ and CO_2_ reduction to CO as a function of operating temperatures are summarized in **Figure** **9**. However, the CO_2_ reduction and product yield under realistic conditions are also governed by factors that affect the CO_2_ reduction kinetics. For example, although the CO_2_ reduction to CH_4_ is thermodynamically favorable at low operating temperatures, the CO_2_ reduction electrode can kinetically favor the CO_2_‐to‐CO conversion, and CO_2_ can be quickly reduced to CO with high selectivity toward CO.^[^ ^28^ ^]^ However, at a high operating temperature, it is challenging to kinetically enhance the CH_4_ production selectivity as a high operating temperature simultaneously favors the steam reforming of CH_4_, which subsequently converts CH_4_ back to CO. Therefore, the intermediate operating temperature of PCECs offers the feasibility and versatility of producing either CO or CH_4_. Additionally, the applied current density and potential also affect the CO_2_ reduction kinetics. CO_2_ reduction to CH_4_ requires more protons than CO production. Therefore, the proton supplied for CO_2_ reduction, which is proportional to the current density, can impact the Faradaic selectivity toward CH_4_ or CO.^[^ ^5^ ^]^

### 3.2 Recent Achievements in Employing PCECs for the CO_2_ Reduction Reaction

**Figure 10:**
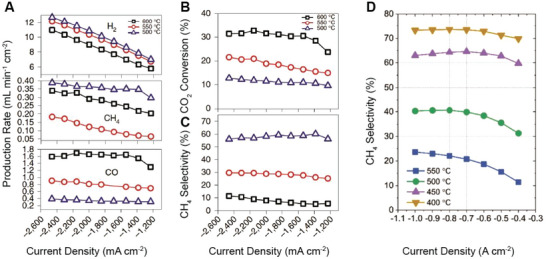
CO_2_ reduction in CO_2_‐PCEC 2 with BaCe_0.7_Zr_0.1_Y_0.1_Yb_0.1_O_3_+Ni (BCZYYb7111+Ni) as the negative electrode. A) Production rate of chemicals produced in a button cell.^[^ ^5^ ^]^ Copyright 2019, The Authors, published by Springer Nature. B) CO_2_ conversion in a button cell. Reproduced under the terms of the Creative Commons Attribution 4.0 International License.^[^ ^5^ ^]^ Copyright 2019, The Authors, published by Springer Nature. C) CH_4_ selectivity in a button cell.^[^ ^5^ ^]^ Copyright 2019, The Authors, published by Springer Nature. D) CH_4_ selectivity in a large area cell (5 cm^2^). Reproduced with permission.^[^ ^98^ ^]^ Copyright 2022, Elsevier.

**Figure 11:**
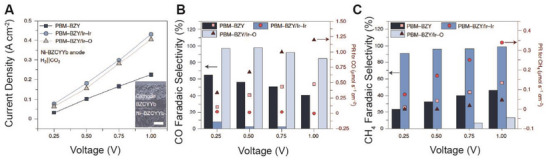
CO_2_ reduction in CO_2_‐PCEC 1 with three different negative electrodes. Reproduced with permission.^[^ ^28^ ^]^ Copyright 2021, Springer Nature. A) I‐V curve of CO_2_ reduction. B) CO Faradaic selectivity and CO production rate. C) CH_4_ Faradaic selectivity and CH_4_ production rate.

Synthesizing chemicals via CO2RR in CO_2_‐PCEC 1 and CO_2_‐PCEC 2 has been experimentally validated. **Figures** **10** and **11** display the most recent demonstrations of PCECs for producing CO and CH_4_. **Table** **3** summarizes additional details in reducing CO_2_ in PCECs. Duan and O'Hayre et al. conducted a preliminary study on reducing CO_2_ in CO_2_‐PCEC 2.^[^ ^5^ ^]^ A composite of BaCe_0.7_Zr_0.1_Y_0.1_Yb_0.1_O_3_ (BCZYYb7111) and Ni was employed as the negative electrode, which enables the conversion of CO_2_ to CO and CH_4_. Figure 10A–C shows BCZYYb7111‐Ni electrode favors HER, although a CO_2_ conversion of >20% has been achieved. The production rates of CO and CH_4_ increase with increasing the operating temperature (Figure 10A,B). Additionally, a high current density enhances the H_2_ utilization/permeation and leads to a higher H_2_/CO_2_ ratio, which is beneficial for increasing the CH_4_ selectivity (Figure 10C). Furthermore, a mixture of CH_4_ and CO is produced, indicating BCZYYb7111‐Ni cannot selectively reduce CO_2_ to either CH_4_ or CO. As shown in Figure 10D, synthesizing CH_4_ in large‐area CO_2_‐PCEC 2 via co‐conversion of CO_2_ and H_2_O has been also validated using BaCe_0.4_Zr_0.4_Y_0.1_Yb_0.1_O_3_ (BCZYYb4411)+Ni as the negative electrode.^[^ ^98^ ^]^ However, the selectivity toward CH_4_ is <80% and a significant portion of the applied current contributes to H_2_ production. Therefore, an optimized negative electrode is needed to inhibit HER and selectively produce one single product. Techno‐economic analysis suggests synthesizing CH_4_ in CO_2_‐PCEC 2 with BCZYYb4411+Ni negative electrode can lead to a levelized cost of fuel production (LCOFP) of $104 MWh^−1^ if the total Faradaic efficiency is increased to >90%, the electricity cost is reduced to $0.02 kWh^−1^, and the H_2_ is recycled for CO_2_ conversion.^[^ ^98^ ^]^ It is expected the CH_4_ production cost can be further reduced if the CO_2_‐to‐CH_4_ selectivity is enhanced to >95%.

**Table 3:** A summary of notable PCECs for CO2RR

Configuration: Positive electrode | Electrolyte | Negative (CO_2_) electrode	T [°C]	Current density [mA cm^−2^]	Voltage [V]	Positive electrode atmosphere	Negative electrode atmosphere	CO_2_ conversion [%]	CO selectivity [%]	CH_4_ selectivity	Total FE [%]	CO Faradaic yield [%]	H_2_ Faradaic yield [%]	CH_4_ Faradaic yield [%]	Stability	Ref.
Ni‐ BaCe_0.5_Zr_0._3Y0.16Zn_0.04_O_3‐*δ*_ |BaCe_0.5_Zr_0._3Y0.16Zn_0.04_8O_3‐*δ*_ |Fe‐ BaCe_0.5_Zr_0._3Y0.16Zn_0.04_O_3‐*δ*_	614	1500	2.60	3% H_2_O+H_2_	CO_2_	65.0	92.5	7.5	35.9	29.5	4.0	2.4	1 h, 1500 mA cm**^−^** ^2^ at 614 °C	[86]
SrEu_2_Fe_1.8_Co_0.2_O_7‐*δ*_+BaZr_0.2_Y_0.8_O_3‐*δ*_| BaZr_0.2_Y_0.8_O_3‐*δ* ‐_|Ni‐ BaZr_0.2_Y_0.8_O_3‐*δ* ‐_	550	0	0.97	10% H_2_O+Air	6% CO_2_+24% H_2_+70% Ar	76.8	89.3	9.9	N/A				95 h, 800 mA cm**^−^** ^2^ at 550 °C	[87]
		100	1.13			75.8	87.6	9.9	22.5	8.4	9.7	4.5		
		200	1.20			74.9	88.0	10.3	32.6	8.7	19.3	4.6		
		400	1.29			72.3	88.8	10.3	42.8	9.6	28.1	5.1		
(La_0.75_Sr_0.25_)_0.95_Mn_0.5_Cr_0.5_O_3‐*δ*_ |BaCe_0.5_Zr_0.3_Y0._16_Zn_0.04_O_3‐*δ*_ |Ni‐ BaCe_0.5_Zr_0.3_Y_0.16_Zn_0.04_O_3‐*δ*_	600	100	2.00	5%H_2_O+Ar	100%CO_2_	N/A	90	10.75	90	90	10	0.0	30 min, 2 V at 600 °C	[89]
La_0.8_Sr_0.2_MnO_3‐*δ*_ – BaCe_0.2_Zr_0.7_Y_0.1_O_3‐*δ*_ | BaCe_0.2_Zr_0.7_Y_0.1_O_3‐*δ*_ |Pt	700	0	0.85	7.5%H_2_O+Ar	9%CO_2_+Ar	15	100	0	N/A	N/A	N/A	0.0	8 h at a current density ranging from 0 to 10.4 mA cm**^−^** ^2^ at 600 °C	[90]
		5	1.25			17.8	100	0	28	5.0	23.0	0.0		
		10	1.62			19.3	100	0	39	7.5	31.5	0		
BaCo_0.4_Fe_0.4_Zr_0.1_Y_0.1_O_3‐*δ*_ | BaCe_0.7_Zr_0.1_Y_0.1_Yb_0.1_O_3‐*δ*_ |Ni‐ BaCe_0.7_Zr_0.1_Y_0.1_Yb_0.1_O_3‐*δ*_	600	1231	N/A	5.5%H_2_O +Air	5.8% CO_2_+ Ar	23.7	95.5	4.5	97.0	22.0	74.0	1.0	N/A	[5]
	550	1231	N/A			15.0	75.9	24.1	97.0	11.1	82.4	3.5		
	500	1231	N/A			9.7	44.8	55.2	97.0	4.2	87.6	5.2		
Ni‐ BaCe_0.7_Zr_0.1_Y_0.1_Yb_0.1_O_3‐*δ*_ | BaCe_0.7_Zr_0.1_Y_0.1_Yb_0.1_O_3‐*δ*_ | PrBaMn_2_O_5+*δ*_+ BaZr_0.7_Y_0.3_O_3−*δ*_	400	32	0.25	100%H_2_	5%CO_2_+Ar	31.4	76.3	23.7	97.0	64.8	8.6	64.8	N/A	[28]
		102	0.50			31.6	67.3	32.7	97.9	56.3	8.9	56.3		
		166	0.75			32.7	60.0	40.0	99.1	50.7	8.4	50.7		
		225	1.00			31.5	53.8	46.2	98.4	40.6	11.7	40.6		
Ni‐BaCe_0.7_Zr_0.1_Y_0.1_Yb_0.1_O_3‐*δ*_ | BaCe_0.7_Zr_0.1_Y_0.1_Yb_0.1_O_3‐*δ*_ | PrBaMn_2_O_5+*δ*_+ BaZr_0.7_Y_0.3_O_3−_ *δ*|Ir‐Ir	400	32	0.25			30.4	9.1	90.9	99.1	8.2	0.1	90.9	100 h, 0.5 V at 400 °C	
		102	0.50			30.5	3.7	96.3	98.8	2.5	0.1	96.3		
		166	0.75			28.5	3.7	96.3	98.5	2.2	0.0	96.3		
		225	1.00			23.7	1.0	99.0	99.0	0.0	0.0	99.0		
Ni‐BaCe_0.7_Zr_0.1_Y_0.1_Yb_0.1_O_3‐*δ*_ | BaCe_0.7_Zr_0.1_Y_0.1_Yb_0.1_O_3‐*δ*_ | PrBaMn_2_O_5+*δ*_+ BaZr_0.7_Y_0.3_O_3−_ *δ*|Ir—O	400	64	0.25			21.6	100.0	0.0	97.3	97.1	0.1	0.0	100 h, 0.5 V at 400 °C	
		156	0.50			20.7	100.0	0.0	98.3	98.2	0.1	0.0		
		281	0.75			20.9	93.4	6.6	99.0	92.3	0.1	6.6		
		405	1.00			18.9	86.9	13.2	98.2	85.0	0.0	13.2		
Ni BaCe_0.7_Zr_0.1_Y_0.1_Yb_0.1_O_3‐*δ*_ BaCe_0.4_Zr_0.4_Y_0.1_Yb_0.1_O_3‐*δ*_ | Sr_2_Fe_1.4_Mo_0.5_O_6‐*δ*_‐Ni_0.175_	600	50	0.07	20%H_2_+N_2_	100%CO_2_	N/A	99.7	0.3	N/A				50 h, 50 mA cm**^−^** ^2^ at 500 °C	[99]
		100	0.07				99.8	0.2						
		150	0.08				99.7	0.3						
		200	0.09				99.7	0.3						
		300	0.11				99.7	0.3						
		400	0.14				99.6	0.4						
	550	50	0.085				99.0	1.0						
		100	0.090				99.4	0.6						
		150	0.010				99.3	0.7						
		200	0.104				99.3	0.7						
		300	0.130				99.3	0.7						
		400	0.147				99.3	0.7						
	500	400	0.159				98.4	1.6						
	450	300	0.152				97.2	2.8						
	400	50	0.108				97.0	3.0						
		100	0.122				97.3	2.7						
		150	0.128				95.1	4.9						
		200	0.140				95.5	4.5						
		300	0.161				96.7	3.3						

To overcome the challenges associated with product selectivity, Ding et al. have demonstrated novel negative electrodes to modulate the selectivity toward CO or CH_4_.^[^ ^24^, ^31^ ^]^ They observed that the interactions between the oxide support and metal clusters can tune the electronic structure of the clusters and consequently affect product selectivity. Both computational modeling and experimental testing reveal that a single Ir atom or a relatively small Ir cluster (Ir—O) on an Sm‐doped CeO_2_ (SDC) surface displays strong interaction and ionic features, which favors CO production. On the other hand, a relatively large Ir cluster (Ir‐Ir) on SDC tends to show metallic features that favor CH_4_ generation. Figures 11A and 10C show the CO_2_‐PCEC 1 with the Ir‐based negative electrode. Both suppressed HER and high selectivity toward either CO or CH_4_ are achieved. For example, as for the PBM‐BZY‐Ir‐Ir catalyst, the CO_2_‐to‐CH_4_ Faradaic selectivity reaches up to 96.3% at 400 °C with an E_bias_ of 0.5 V. On the other hand, under the same operating conditions, PBM‐BZY/Ir—O favors the CO_2_‐to‐CO production, with a CO_2_‐to‐CO Faradaic selectivity of 98.2% achieved, which suggests modulating the negative electrode can tune the selectivity while suppressing HER.

As CO2RR in PCECs is an emerging technology, as shown in Table 3, prior work has focused on performing proof‐of‐concept or enhancing the product yield, while fewer efforts were devoted to enhancing the selectivity of the desired product and inhibiting hydrogen evolution reaction.^[^ ^20^, ^22^, ^31^, ^75^, ^76^, ^77^, ^78^, ^79^ ^]^ Therefore, the selectivity of CO2RR in PCECs is summarized here to identify challenges and offer suggestions to address them.

**Figure 12:**
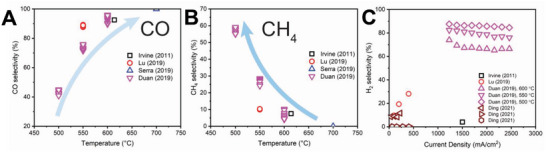
The selectivity of CO_2_ reduction toward CH_4_ and CO in PCECs has been recently demonstrated.^[^ ^5^, ^28^, ^86^, ^87^, ^90^ ^]^ A) The selectivity of CO as a function of operating temperature. B) The selectivity of CH_4_ as a function of operating temperature. C) The Faradaic selectivity of H_2_ as a function of operating temperature.

The selectivity of CO_2_ reduction in PCECs is shown in **Figure** **12**A,B. The selectivity toward methane tends to increase with reducing the operating temperatures while high operating temperatures favor CO production, which is consistent with the thermodynamics of CO2RR, indicating that CO_2_ reduction in PCERs is thermodynamically controlled. As CH_4_ is thermodynamically stable at low temperatures while CO is thermodynamically stable at high temperatures. Therefore, the products synthesized are primarily controlled by reaction thermodynamics rather than kinetics. At relatively low operating temperatures (e.g., <400 °C), CH_4_ is thermodynamically stable, and thus CH_4_ production is more favorable. A CO_2_ reduction electrode that reduces the activation energy of CH_4_ production can further kinetically facilitate the production of CH_4_. However, to enable selective CO_2_‐to‐CO production, the CO_2_ reduction electrode should be rigorously designed to significantly reduce the CO_2_‐to‐CO reaction activation energy and increase the CO production at a faster speed than that of CH_4_ production, which will give rise to a high selectivity toward CO. At an operating temperature of around 600 °C, the thermodynamic stability of CO and CH_4_ are similar. The selectivity cannot be thermodynamically controlled and both CO and CH_4_ can be easily produced. To enhance the selectivity toward either CO or CH_4_, the kinetics of corresponding reactions should be controlled to accelerate the speed of producing a target product. However, due to the high operating temperature, CH_4_ can be converted back to CO via steam reforming. Therefore, the negative electrode should also be designed to suppress the steam methane reforming.

Figure 12C presents the Faradaic selectivity toward hydrogen as a function of current density, implying the HER concurrently occurs with the CO_2_ reduction. Without using the CO_2_ reduction electrode that is rationally designed for PCECs, HER is the predominant reaction in PCECs that are equipped with conventional Ni‐based CO_2_ reduction electrodes (Duan 2019 in Figure 12C).^[^ ^5^ ^]^ Furthermore, the HER tends to be more significant at a high current density as the surface hydrogen or proton cannot be quickly utilized for CO_2_ reduction. The PCECs with Ir‐based CO_2_ reduction electrode deliver a H_2_ Faradaic selectivity <10% (Ding 2021 in Figure 12C), which efficiently and selectivity produce either CH_4_ or CO at a current density <400 mA cm^−2^.^[^ ^24^, ^28^ ^]^ These achievements suggest both the operating conditions, including temperature and current density, and the properties of the CO_2_ reduction electrode control the selectivity toward a specific product.

### 3.3 Mechanisms of CO_2_ Reduction in PCECs

A detailed understanding of the CO_2_ reduction mechanisms will open the “black box” of CO2RR in PCECs. Due to the lack of appropriate tools to conduct in situ operando experiments and probe the active intermediates involved in CO_2_ reduction, the CO_2_ reduction mechanisms in PCECs are not illuminated. Establishing this understanding, especially by leveraging the thermochemical knowledge,^[^ ^88^, ^89^ ^]^ will therefore assist in rationally designing CO_2_ reduction catalysts that could be applied to PCECs.

**Figure 13:**
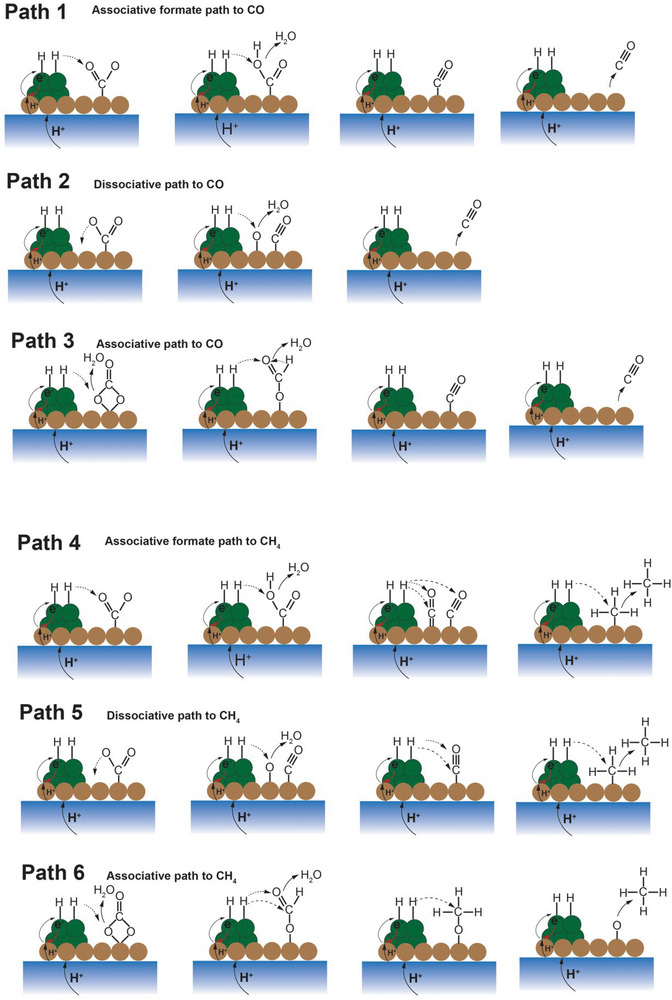
Schematic illustration of the proposed CO2RR mechanisms in PCECs. The blue rectangle is the proton‐conducting electrolyte membrane. The brown dots are the oxide supports and the green dots are metallic particles, which act as the CO_2_ electrode.

**Figure** **13** displays the proposed CO_2_ reduction pathways, including the associative CO_2_ reduction pathways and dissociative pathways. The associative pathways denote the pathways where the C—O bond cleavage occurs after its hydrogenation while the dissociative pathways represent the C—O bond will first break and then be hydrogenated. The CO_2_ reduction pathway could also be divided into two categories depending on the product, which is CO or CH_4_. Despite other hydrocarbons, such as ethylene and life olefins, could be produced in PCECs that operate at an intermediate temperature at which the C—C bond coupling is thermodynamic favorable, this concept has not been experimentally validated. Thus, the CO_2_‐to‐light olefins mechanisms are not discussed in this review. As shown in Figure 13, the protons from the positive electrode could first pair with electrons to form hydrogen adsorbed on the negative electrode (*H). The negative electrode is typically a composite of oxide support and a metallic phase. The proton is therefore reduced at the oxide‐metal interface and the hydrogen will then adsorb on the surface of the metallic phase. It will subsequently reduce the intermediate species adsorbed on the oxide. The potential CO_2_ reduction pathways are outlined as follows:

Path 1: CO_2_ is associatively adsorbed on the oxide surface and forms carbonate that will be then reduced to formate. The formate species will be further reduced to CO.

Path 2: Oxygen vacancies are available on the oxide support. One oxygen atom in a CO_2_ molecule occupies the oxygen vacancy. CO_2_ will then be dissociatively adsorbed on the oxide surface. The resultant CO will desorb and produce CO.

Path 3: CO_2_ molecules associatively adsorb on the negative electrode followed by the formation of bidentate carbonate. The bidentate carbonate will be subsequently hydrogenated to bicarbonate which can be further reduced to CO.

Path 4: This path displays the associative formate path for CH_4_ production. CO_2_ molecules associatively adsorb on the negative electrode and form formate species that can be subsequently reduced to CH_4_.

Path 5: CO_2_ molecules dissociatively adsorb on the negative electrode and produce *CO that will be consecutively reduced to CH_4_.

Path 6: CO_2_ molecules associatively adsorb on the negative electrode and produce formate that will be further reduced to CH_4_.

### 3.4 Challenges Associated with CO2RR in PCECs

**Figure 14:**
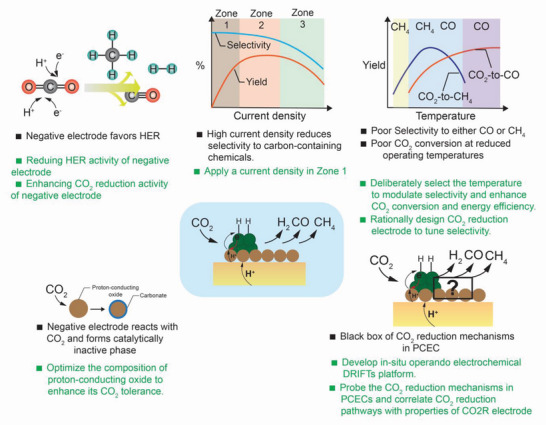
Summary of challenges and future perspectives to improve the chemical synthesis in PCECs via CO2RR.

According to the current achievements of CO_2_ reduction that are summarized in Figures 10, 11, 12, and the PCEC performances have been demonstrated,^[^ ^20^, ^22^, ^28^, ^86^, ^87^, ^88^, ^89^, ^90^ ^]^ we have identified the challenges of converting CO_2_ in PCECs. As shown in **Figure** **14**, these challenges include 1) high H_2_ selectivity; 2) poor Faradaic selectivity to either CO or CH_4_; 3) poor chemical stability; and 4) the lack of established CO_2_ reduction mechanisms. These challenges are detailed as follows:

As PCECs for CO_2_ conversion is a truly nascent technology, most previous studies were performed directly using the negative electrode of protonic ceramic fuel cell (PCFC), which is a composite of proton‐conducting oxide and Ni,^[^ ^5^, ^20^ ^]^ as the CO_2_ reduction electrode. Ni‐based cermet also favors HER. Therefore, the Faradaic selectivity toward carbon‐containing molecules is poor. Additionally, oxide‐supported Ni is not prone to selectively produce either CO or CH_4_. Thus, the product is commonly a mixture of H_2_, CO, and CH_4_. Furthermore, the negative electrode that favors HER leads to poor CO_2_ conversion.

Oxygen‐ion solid oxide electrochemical cells (O‐SOECs) have been widely used for efficiently producing syngas via co‐electrolysis of CO_2_ and H_2_O or producing pure CO via CO_2_ electrolysis.^[^ ^102^ ^]^ However, the products are limited to either syngas or CO. As the operating temperature of PCECs is lower than that of O‐SOECs, it is feasible to produce CH_4_.^[^ ^5^, ^24^, ^28^ ^]^ However, this competitive advantage also poses challenges in selectively producing high‐purity chemicals. The selection of negative electrode materials will determine the Faradaic selectivity. There is a lack of rationally designed negative electrode materials that can lead to the production of pure CO, syngas, or CH_4_. To selectively produce pure CO or CH_4_, the negative electrode should also significantly suppress the HER.

One of the innovations of CO_2_ reduction in PCECs is that PCECs can operate at a lower temperature than O‐SOECs. However, as the CO_2_ molecules are thermodynamic stable, the CO_2_ reduction kinetics is slower than that at high operating temperatures, especially for CO production. Therefore, the CO_2_ conversion to CO in PCECs is lower than that in O‐SOECs. The intermediate operating temperatures thermodynamically favor CO_2_ reduction to hydrocarbons. Therefore, it is suggested future research should be geared toward enhancing the electrocatalytic activity of negative electrodes at intermediate operating temperatures (e.g., 300–500 °C) to improve CO_2_ conversion to hydrocarbons.

One of the impediments to the development of commercially relevant PCECs is the long‐term durability of PCECs, especially the negative electrode under an atmosphere with CO_2_. Reduced operating temperatures thermodynamically favor the CO_2_ reduction to hydrocarbons. However, lowering the operating temperature may compromise the chemical stability of negative electrodes that are more prone to react with CO_2_ and form BaCO_3_ at low temperatures. The BaCO_3_ phase is an insulator and catalytically inert, leading to the degradation of PCECs.^[^ ^2^, ^86^ ^]^

Despite coking (or carbon deposition) not being observed in PCECs, it does not indicate coking is not possible as the current CO_2_ conversion in PCECs is poor. The future PCECs developed for CO_2_ reduction to CO could achieve a high CO/CO_2_ ratio. The intermediate operating temperature further favors the exothermic Boudouard reaction, posing potential risks of coking.^[^ ^103^ ^]^

The CO_2_ reduction mechanism in PCECs is the “black box”. It is widely accepted that CO_2_ is reduced by either the proton or the gaseous H_2_. However, there is no direct evidence of how electrocatalysis occurs and what intermediate species are involved in the reactions. The lack of this fundamental understanding hinders the rational design of negative electrodes for PCECs. Therefore, without understanding the CO_2_ reduction pathway over negative electrodes, it is impossible to deliberately modulate the negative electrode composition and structures to tune the CO_2_ reduction pathways.

### 3.5 Strategies to Address the Challenges of CO_2_ Conversion and Utilization in PCECs

Figure 14 displays the challenges of synthesizing chemicals in PCECs via CO_2_ reduction and corresponding strategies. There is a variety of strategies that can tackle these problems, which include 1) designing CO_2_ reduction electrodes for PCECs to inhibit HER, enhance CO2RR activity, and tune the CO_2_ reduction pathways for enhancing the propensity to produce desired products; 2) enhancing chemical stability of CO_2_ reduction electrode by improving the CO_2_ tolerance of proton‐conducting oxide support; 3) alternating the operating conditions to modulate selectivity while achieving high CO_2_ conversion and product yield; and 4) conducting in situ operando experiments to probe the CO2RR mechanisms in PCECs. These strategies can also be intertwined to better control the selectivity and further enhance CO_2_ conversion.

#### 3.5.1 Inhibit Hydrogen Evolution Reaction

HER is thermodynamically more favorable than CO2RR, suggesting HER can only be inhibited by modulating the kinetics of HER and CO2RR on the negative electrode. Two sets of strategies could be applied to suppress HER, which include reducing the HER activity of the negative electrode and improving its CO2RR kinetics. The negative electrode is typically a composite of transition metal and oxide. The transitional metal, including Ni, Co, Cu, and Fe, is key to the HER activity. Although there is limited study of the HER activity on transition metals in gaseous media, it has been recognized that the intrinsic properties of transition metals alternate the HER activity. For example, in acidic media, the HER activity of the most common transition metals shows the following trend: Ni>Co>Cu>Fe, which suggests tailoring the transition metal can inhibit the HER.^[^ ^104^ ^]^ Additionally, an Ir‐based negative electrode has been applied as the PCEC negative electrode for CO_2_ reduction, which significantly inhibits the HER without compromising the CO2RR activity, validating the metallic phase is essential for HER.^[^ ^28^ ^]^ However, in acidic media, HER on Ir is more favorable than that on Ni.^[^ ^104^ ^]^ Therefore, the volcano plot developed for HER in acidic media cannot be directly used to develop CO_2_ reduction electrodes for PCECs, suggesting the HER activity on transition metal in gaseous media should be better studied.^[^ ^105^, ^106^, ^107^ ^]^

#### 3.5.2 Faradaic Selectivity toward CO or CH_4_

Thermochemical CO_2_ reduction over heterogeneous catalysts has been well studied, establishing strategies to tune the selectivity toward CO and CH_4_. Despite the electrochemical CO_2_ reduction pathways in PCECs being different from thermochemical CO_2_ reduction, the approaches to creating and modulating the active intermediate species are similar. Therefore, the strategies established for tuning the selectivity of thermochemical CO_2_ reduction could be potentially applied for tailoring the CO_2_ reduction electrode of PCECs, especially the approaches to selecting a metallic phase.^[^ ^108^, ^109^, ^110^ ^]^

For instance, Ni—Co bimetallic heterogeneous catalysts have been widely used for thermochemical CO_2_ methanation because cobalt is the most active metal for methanation reaction and the Ni—Co alloy (e.g., Co/Ni = 1) can lead to synergistic effects which can significantly enhance the catalytic activity and CH_4_ yield.^[^ ^111^, ^112^, ^113^, ^114^, ^115^ ^]^ To selectively produce CO, using Ni‐free nanoparticles, such as Fe—Co alloy nanoparticles, could be a plausible approach. Fe tends to absorb CO_2_ and can selectively produce CO. However, the activation energy to produce CO is high and thus its overall catalytic activity is low. Fe—Co alloy with a Fe/Co ratio of >3 can drastically enhance its catalytic activity while it does not reduce the CO selectivity because 25 mol% Co can decrease the CO_2_‐to‐CO reaction activation energy.^[^ ^116^ ^]^

The oxide support is also essential for CO_2_ reduction as it typically functions as the site for forming active species, including carbonate, hydroxyl groups, formate, and other functional groups. However, the oxide support developed for thermochemical CO_2_ reduction, such as Al_2_O_3_, TiO_2_, and SiO_2_, might not be directly used for PCECs as they are not conductive. The negative electrodes of PCECs should have electrocatalytic interfaces that are the triple‐phase boundaries, namely the junctions of metal, oxide support, and gas phases. The oxide support should be a mixed ionic and electronic conductor to create the electrocatalytic interfaces.

#### 3.5.3 Improve Chemical Stability of Negative Electrode

The chemical stability CO_2_ reduction electrode is key to the durable operation of PCECs, which depends on the CO_2_ tolerance of the oxide phase (i.e., proton‐conducting oxide). Therefore, the approaches developed to enhance the CO_2_ tolerance of proton‐conducting oxides or related materials can be leveraged. Herein, we focus on reviewing the proton‐conducting oxide with a generalized formula of ABO_3_ as it is the most widely used oxide in PCECs. Its chemical stability could be improved by doping the A‐site or B‐site with other cations that inhibit its chemical reaction with CO_2_. For example, doping the A‐site with Ca and La can increase CO_2_ tolerance. Moreover, it has been recognized that increasing the amount of Zr at the B‐site also leads to improved chemical stability.^[^ ^117^, ^118^, ^119^, ^120^, ^121^ ^]^

#### 3.5.4 Establish the Experimental Tools to Probe the CO2RR Mechanisms in PCECs

Though previous studies have attempted to electrochemically reduce CO_2_ in PCECs by integrating it with water electrolysis or dehydrogenation of hydrocarbons, it is not fully clear if the CO_2_ conversion pathway involves any electrochemical processes, as CO_2_ reduction in negative electrodes may proceed through either electrochemical pathway or thermochemical pathway, or both.

Therefore, complementing the electrochemical cell design, CO_2_ reduction electrode design, fabrication, and characterization, it is important to probe and understand the CO2RR mechanisms and establish the relationship between CO_2_ reduction mechanisms and electrode materials. This fundamental understanding will allow us to establish strategies to deliberately design the CO_2_ reduction electrode. The active functional groups involved in CO_2_ reduction are not systematically studied and correlated with the CO_2_ reduction electrode. Without a fundamental understanding of CO_2_ reduction pathways, it is unfeasible to deliberately design and fabricate highly active CO_2_ reduction electrodes. It is essential to establish the in situ operando spectroscopy technologies, such as in situ operando Diffuse Reflectance Infrared Fourier Transform Spectroscopy (DRIFTS), to probe the intermediates and better understand the CO2RR mechanisms.

The in situ operando DRIFTS platform is designed for studying thermochemical heterogeneous catalysis,^[^ ^80^, ^122^, ^123^, ^124^ ^]^ which cannot directly apply a potential on the samples. The in situ operando DRIFTS chamber available on the market should be optimized or customized to allow performing in situ operando electrochemical DRIFTS. The establishment of this platform will advance the fundamental study of electrochemical CO2RR mechanisms in PCECs.

As the choice of proton‐conducting oxide support and metallic phase in negative electrodes can significantly impact CO2RR mechanisms, the factors that have relations with the CO2RR mechanism include the support, metallic phase, and the intertwining effects between support and metallic phase.^[^ ^125^ ^]^ It is expected that different supports, metals, and the combination of support and metal could result in distinct intermediate species, which consequently leads to different CO2RR pathways, reaction kinetics, and selectivity. Therefore, the in situ operando electrochemical DRIFTS is suggested to be utilized to thoroughly illuminate these impacts, which can provide extensive guidance on designing CO_2_ reduction electrode materials.

#### 3.5.5 Adjust Operating Conditions to Modulate the Product Yield, Selectivity, and CO_2_ Conversion

The operating temperature has a huge impact on CO_2_ reduction kinetics and CO_2_ conversion. Therefore, the operating temperature should be rigorously selected considering both CO_2_ reduction kinetics over the negative electrode and CO_2_ conversion. In general, to produce CO with a high CO_2_ conversion and energy efficiency, the negative electrode should be designed to kinetically favor CO production while operating PCECs at an appropriately high temperature. To selectively produce CH_4_, the negative electrode should be engineered to reduce CO_2_ to CH_4_ and PCECs should operate at relatively low temperatures (e.g., 400 °C) to further enhance CH_4_ production. It is also essential to keep in mind that reducing the operating temperature can certainly enhance the overpotential of oxygen evolution reaction at positive electrodes. The production of CH_4_ could be enhanced by reducing operating temperature, but at the cost of reduced energy efficiency.

Furthermore, the applied current density is the second factor that affects the CO_2_ reduction in PCECs as the current density is proportional to the proton flux and hydrogen concentration in the negative electrode. Therefore, adjusting the current density will affect the CO_2_ reduction performances. It has been noted that electrochemical promotion does exist for ammonia synthesis (nitrogen reduction reaction) after integrating an ammonia synthesis catalyst with a proton‐conducting ceramic membrane.^[^ ^48^ ^]^ The electrochemically promoted ammonia production rate is 13 times higher than the thermochemical ammonia production rate while the proton consumed for ammonia synthesis is six times higher than that of electrochemically supplied, indicating a significant non‐Faradaic impact exists which is attributed to the enhanced proton concentration on the catalyst surface. Therefore, we anticipate that similar electrochemical promotion also plays a role in CO2RR because the CO2RR is also mediated by the local proton/hydrogen concentration on the catalyst surface.^[^ ^26^, ^126^ ^]^ Referring to Figures 7 and 14, the Faradaic selectivity toward carbon‐containing chemicals as a function of the current density should display a similar relationship, implying that increasing the applied current density cannot continuously enhance CO_2_ conversion, product yield, and Faradaic selectivity. Therefore, an appropriately high current density, as shown in Zone 1 of Figure 14 (Top middle panel), could concurrently achieve a high product yield and selectivity.

Finally, we would like to emphasize that PCECs provide versatility in terms of operating conditions and products. There is no straightforward principle of selecting the optimal operating conditions, which should be carefully determined after considering the chemical to be produced, selectivity, CO_2_ conversion, and energy efficiency, especially when those factors are considered in the techno‐economic analysis.

## 4 Upgrading Natural Gas in PCECs

### 4.1 Motivations of Employing PCECs for Distributed Natural Gas Conversion

PCECs offer several intriguing advantages for upgrading natural gas to value‐added chemicals and provide potential opportunities for distributed chemical production. First, we provide a brief overview of the industrial context that stimulates the research of employing PCECs for upgrading natural gas.

The depletion of conventional oil reserves has shifted much oil production to remote and offshore reservoirs, which imposes severe restrictions on the utilization of co‐produced natural gas. Facilities for natural gas conversion rarely exist onsite, while transporting gas to centralized facilities is cost‐prohibitive and/or infeasible. Consequently, flaring and venting are commonly used to dispose of co‐produced natural gas. Nonetheless, both practices flagrantly waste precious resources with deleterious environmental impacts.

**Figure 15:**
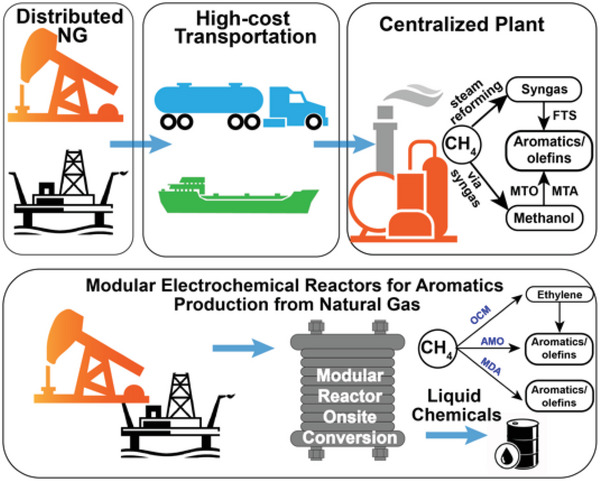
Pathways of synthesizing chemicals from natural gas/methane. NG: natural gas; FTS: Fischer–Tropsch synthesis; MTO: methanol to olefins; MTA: methanol to aromatics; OCM: oxidative coupling of methane; AMO: Aromatization of methane under oxidizing atmosphere; MDA: methane dehydroaromatization.

Efficient and economical utilization of flared and vented natural gas motivates the development of distributed and modular technologies for producing liquid or solid chemicals to enable economical transportation and utilization of the resulting value‐added products.^[^ ^127^, ^128^ ^]^ **Figure** **15** summarizes the pathways of synthesizing chemicals from methane (the primary component of natural gas). Currently, the syngas and Fischer–Tropsch synthesis route is the most mature technology for converting natural gas to commodity chemicals (Figure 15, top panel). However, this centralized technology is not suitable for onsite chemical production because it is a complicated process that is difficult to downsize and requires costly infrastructure to transport natural gas.^[^ ^129^ ^]^ Additionally, Fischer–Tropsch synthesis consumes either extra CO or H_2_ to remove the oxygen from CO, leading to lower carbon utilization or consumption of valuable H_2_.^[^ ^130^ ^]^ Alternatively, syngas can be converted to methanol followed by methanol‐to‐olefins conversion^[^ ^131^ ^]^ or methanol‐to‐aromatics conversion.^[^ ^132^ ^]^ However, this technology also relies on energy‐intensive and centralized syngas production.

There is an urgent need for modular and distributed chemical production systems (Figure 15, bottom panel) that can directly, efficiently, and economically convert natural gas to high‐value and transportable chemicals. It should be noted that the global demand for benzene has reached about 43 million tonnes in 2020.^[^ ^133^ ^]^ Moreover, the U.S. imports 20% of all the benzene it uses because of a shortage of benzene supply.^[^ ^133^ ^]^ These facts are driving the industry toward producing aromatic compounds directly from methane via nonoxidative methane dehydroaromatization (MDA), oxidative methane aromatization, or oxidative coupling of methane to ethylene followed by further oligomerization to aromatics.^[^ ^134^, ^135^, ^136^, ^137^, ^138^ ^]^

Direct, nonoxidative conversion of methane to aromatics has attracted widespread attention because it enables high‐value liquid chemicals production, eliminates complex reactors and processes, and avoids methane overoxidation, as well as significantly reduces CO_2_ emissions.^[^ ^138^ ^]^ However, the nonoxidative MDA over heterogeneous catalysts in conventional packed bed reactors is challenging because of its low methane conversion (typically ≈10% at 750 °C),^[^ ^139^ ^]^ coking, and the high operating temperatures (>800 °C) for C—H bond activation.^[^ ^138^, ^139^, ^140^, ^141^, ^142^ ^]^ Although a high methane conversion of 48.1% and a stable operation exceeding 60 h have been demonstrated over single‐atom iron sites embedded in a silica matric, its operating temperature is impractically high (>1000 °C), and the selectivity of the aromatic compounds is low (<50%).^[^ ^138^ ^]^ In the last decades, a host of technologies have been proposed for addressing these challenges. These include injecting oxygen to mitigate coke and improve durability, and extracting hydrogen to circumvent the severe thermodynamic limitations and thereby enhancing methane conversion and product yield.^[^ ^143^, ^144^, ^145^, ^146^ ^]^ Unfortunately, it is infeasible to achieve simultaneous hydrogen extraction and oxygen injection in packed bed reactors to tackle all the above challenges.

**Figure 16:**
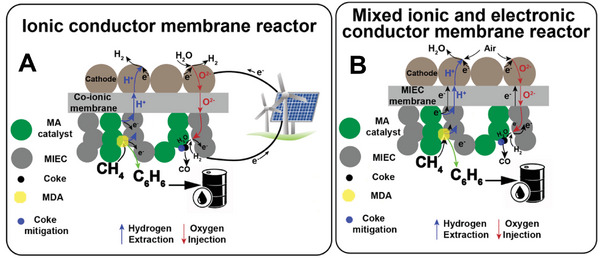
Schematic illustration of PCECs for producing aromatics from natural gas. A) PCECs with a mixed proton and oxygen‐ion conductor as the membrane. B) PCECs with a mixed proton, oxygen‐ion, and electronic conductor as the membrane. MA: methane aromatization; MIEC: mixed ionic and electronic conductor; MDA: methane dehydroaromatization.

As an alternative strategy, PCECs (**Figure** **16**),^[^ ^1^, ^5^, ^23^, ^127^ ^]^ which can operate in power‐driven (electrolytic reactor, Figure 16A) mode and hydrogen permeation mode (Figure 16B), enable continuous and high‐flux hydrogen extraction, and minor oxygen injection. The hydrogen extraction aims to enhance methane conversion and aromatics yield while minor oxygen injection can improve its coking tolerance. Figure 16A displays the PCECs with a mixed proton and oxygen‐ion conductor as the membrane, which extracts H_2_ and injects minor O_2_ under an external potential. Figure 16B shows the PCECs that employ a mixed proton, oxygen‐ion, and electronic conductor as the membrane. The chemical potential drives H_2_ permeation and oxygen injection.

The oxygen‐ion conduction of the membrane is key to durability. Additionally, the transference number of oxygen ions can affect the selectivity of products. A relatively low oxygen‐ion transference number can mitigate coking while selectively producing aromatics. However, an excessively high oxygen‐ion conduction can oxidize the hydrocarbons and reduce the selectivity of aromatics. Therefore, the transference number of oxygen ions should be controlled at lower than 10% to simultaneously achieve durable operation and high aromatics yield. It has been widely recognized that the operating temperature and atmosphere can alter the transference number of oxygen ions.^[^ ^5^, ^49^, ^147^ ^]^ In general, increasing the operating temperature and oxygen partial pressure can increase the transference number of oxygen ions. Duan and O'Hayre et al. also noted BaCe_0.7_Zr_0.1_Y_0.1_Yb_0.1_O_3‐𝛿_ exhibits a higher transference number of oxygen ions than BaZr_0.8_Y_0.2_O_3‐𝛿_, indicating the electrolyte composition also affects the oxygen‐ion injection.^[^ ^147^ ^]^ As one of the benefits of employing PCECs for producing aromatics is the reduced operating temperature, increasing the temperature to achieve a relatively high transference number of oxygen ions is not favorable. However, the oxygen partial pressure of the cathode side or electrolyte composition can be adjusted to control the oxygen‐ion injection.

Incorporating deliberately designed methane aromatization catalysts into PCECs and synergizing the mixed proton and oxygen‐ion conduction address the two main issues of methane aromatization in conventional thermochemical catalytic processes: 1) low methane conversion due to thermodynamic limitations and 2) rapid catalyst deactivation due to coking. By continually removing hydrogen (blue arrows in Figure 16) from the methane aromatization electrode side, this hybrid process pushes the reaction equilibrium toward aromatics, thereby increasing methane conversion (Figure 16, yellow dot) at reduced temperatures (e.g., 600 °C).^[^ ^142^, ^148^ ^]^ Second, the electrolyte membrane exhibits minor oxygen‐ion conductivity,^[^ ^49^, ^147^ ^]^ which enables the concomitant injection of oxygen (red arrows in Figure 16) to the methane aromatization electrode which can act to burn off coke or selectively react with H_2_ and hydrocarbons to produce H_2_O and CO_2_ which can then mitigate coking (blue dot in Figure 16).

A computational study^[^ ^136^ ^]^ found that the addition of an appropriate amount of oxygen facilitates methane aromatization by the formation of ethylene via oxidative coupling of methane followed by the aromatization of ethylene. Cao et al.^[^ ^137^ ^]^ have experimentally demonstrated oxidative methane aromatization in mixed oxygen‐ion and electronic membrane reactors. Although enhanced stability and inhibited coke formation were achieved in the reactor, the aromatics yield, in particular the initial aromatics yield, was not significantly enhanced.^[^ ^137^ ^]^ In conventional fixed bed rectors, there have been attempts to regenerate the methane aromatization catalyst by burning out the carbon using oxygen at 700 °C.^[^ ^149^, ^150^, ^151^ ^]^ However, due to relatively high oxidation temperature, Mo_2_C, the active site on methane aromatization catalysts, tends to be reoxidized into mobile Mo‐oxides (i.e., overoxidation). Therefore, the regeneration of catalysts at lower temperatures (500–600 °C) has been employed to reactivate the catalysts.^[^ ^143^, ^152^ ^]^ However, temperature cycling in a fixed‐bed reactor is inefficient and complex.

Extracting a considerable amount of hydrogen to shift reaction equilibrium has been achieved in PCECs to enhance methane conversion and aromatics yield.^[^ ^145^, ^146^ ^]^ The potential of PCECs for improving methane aromatization has been validated.^[^ ^23^ ^]^ If MDA can run at lower temperatures, coke formation will be suppressed since the formation of polyaromatic type carbon, the main reason for catalyst deactivation, is more prone to occur at high temperatures.^[^ ^142^, ^148^ ^]^ Liu et al. also recognized that reduced operating temperatures will inhibit coke formation.^[^ ^153^ ^]^ However, further dehydrogenation favors undesired naphthalene and thus will lead to its oligomerization to polyaromatic hydrocarbons, which are the deleterious coking species.^[^ ^139^, ^145^ ^]^ Rival et al. noted that dehydrogenation via the Pd‐alloy membrane reactor results in more severe graphitic coke.^[^ ^145^ ^]^ Therefore, although enhanced methane conversion was achieved in hydrogen‐permeation membrane reactors (e.g., Pd and Pd‐alloy membrane reactor), the exacerbated coking makes it impractical for economically viable methane conversion.

**Figure 17:**
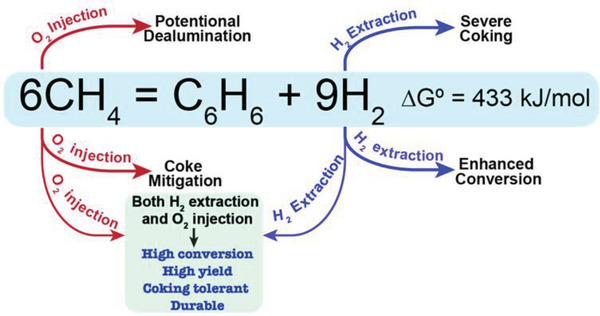
Impacts of H_2_ extraction and O_2_ injection on methane aromatization.

In summary, either hydrogen extraction or oxygen injection exhibits both beneficial and detrimental impacts, which are summarized in **Figure** **17**, implying the challenges of methane aromatization cannot be solely addressed via hydrogen extraction or oxygen injection. As depicted in Figure 17, it is expected that the integration of hydrogen extraction with oxygen injection could potentially address all issues of low conversion, low yield, and poor durability. Thanks to the unique defect chemistry and transport properties of the protonic ceramic electrolyte (e.g., BaCe_x_Zr_y_Y_0.1_Yb_0.1_O_3‐*δ*_, x+y = 0.8), simultaneous hydrogen extraction and oxygen injection can be easily achieved in PCECs. **Table** **4** summarizes the amalgamated strategies employed by the PCECs to tackle the two primary challenges of methane aromatization. Therefore, we fully anticipate PCECs will be a significant step toward producing aromatics from distributed natural gas.

**Table 4:** Summary of combined strategies employed in PCECs to address the challenges of upgrading natural gas via methane aromatization

Approaches and strategies	Challenge 1: Low methane conversion	Challenge 2: Severe coking and fast degradation
Hydrogen extraction via proton conduction	Hydrogen extraction drives the reaction toward a higher methane conversion	Hydrogen extraction lowers the operating temperatures to inhibit coking^[^ ^142^, ^148^ ^]^
Oxygen injection via oxygen‐ion conduction	Oxygen injection leads to oxidative methane aromatization and oxidative coupling of methane, which are thermodynamically favorable reactions and thus can enhance methane conversion^[^ ^137^ ^]^	Oxygen injection mitigates coking via direct burn‐off coke, selectively oxidation of Mo_2_C followed by the Mo_2_O_3_‐catalyzed coke mitigation, or the formation of intermediates (H_2_O, CO_2_) which can mitigate both graphitic and polyaromatic coke^[^ ^143^ ^]^
Natural gas as the feedstock. Direct natural gas conversion	C2‐C4 alkanes in natural gas can make MDA thermodynamically more favorable^[^ ^154^ ^]^	Natural gas contains sulfur. The membrane has excellent sulfur tolerance.^[^ ^1^ ^]^ Sulfur will not lead to exacerbated degradation

### 4.2 Recent Demonstrations of Upgrading Natural Gas in PCECs

Converting methane to aromatics in PCECs has centered on developing PCECs in either planar or tubular geometry.^[^ ^23^, ^78^, ^155^, ^156^ ^]^ The most recent demonstrations of employing PCECs and protonic ceramic membrane reactors are summarized in **Table** **5**. Tubular PCECs are more favorable as they can achieve a longer gas stream residence time than the planar PCECs, consequently enhancing the hydrogen extraction rate, methane conversion, and aromatic yield. According to the membrane that is used for building PCECs, the two primary PCEC configurations shown in Figure 16 have been developed. Herein, we focus on reviewing the PCECs that fall into these two categories.^[^ ^23^, ^157^, ^158^ ^]^

**Table 5:** A summary of PCECs for upgrading natural gas

PCEC Application	PCEC Configuration	T [°C]	P [atm]	Current density [mA cm^−2^]	Voltage [V]	Reactants Composition	Conversion [%]	Selectivity [%]	Stability	Ref.
PCEC for ethane deprotonation	BaCe_0.7_Zr_0.1_Y_0.1_Yb_0.1_O_3‐*δ*_‐Ni (Ethane electrode) | BaCe_0.7_Zr_0.1_Y_0.1_Yb_0.1_O_3‐*δ*_ (Electrolyte)|PrBa_0.5_Sr_0.5_Co_1.5_Fe_0.5_O_5+*δ*_ (Hydrogen evolution reaction electrode)	400	1	1000	0.41	10% C_2_H_6_/Ar	2.8 (Ethane)	100 (Ethylene)	90 h, 400 °C, and 1000 mA cm**^−^** ^2^	[25]
PCEC for ethane deprotonation	BaCe_0.7_Zr_0.1_Y_0.1_Yb_0.1_O_3‐*δ*_‐(PrBa)_0.95_(Fe_0.9_Mo_0.1_)_2_O_5+*δ*_ ‐PtGa/ZSM‐5 (Ethane electrode) | BaCe_0.7_Zr_0.1_Y_0.1_Yb_0.1_O_3‐*δ*_ (Electrolyte)| BaCe_0.7_Zr_0.1_Y_0.1_Yb_0.1_O_3‐*δ*_ ‐Ni (Hydrogen evolution reaction electrode)	550	1	0	N/A	10% C_2_H_6_/Ar	21.7 (Ethane)	81.4 (Ethylene)	150 h, 550 °C, and 40 mA cm**^−^** ^2^	[157]
				10			25.9 (Ethane)	79.3 (Ethylene)		
				20			30.6 (Ethane)	77.6 (Ethylene)		
				30			36.1 (Ethane)	72.1 (Ethylene)		
				40			39.9 (Ethane)	66.6 (Ethylene)		
PCEC for methane upgrading	Cu‐Mo/H‐MCM‐22(Methane electrode) | BaZr_0.7_Ce_0.2_Y_0.1_O_3–*δ*_ (Electrolyte)|Ni‐BaZr_0.7_Ce_0.2_Y_0.1_O_3–*δ*_ (Hydrogen evolution reaction electrode)	710	1	40	N/A	10% CH_4_/H_2_	11.6 (Methane)	86.2 (Aromatics)	40 h, 710 °C, and 40 mA cm**^−^** ^2^	[23]
Proton membrane reactor for methane upgrading	Fe‐SiO_2_+SrCe_0.8_Zr_0.2_O_3–*δ*_ (Methane side) | SrCe_0.7_Zr_0.2_Eu_0.1_O_3–*δ*_ (Membrane)	980	1	N/A		90% CH_4_/Ar	5.9 (Methane)	42.9 (Aromatics)	50 h, 1030 °C, and 1 atm	[158]
		1000					10.1 (Methane)	46.4 (Aromatics)		
		1030					18.2 (Methane)	47.4 (Aromatics)		
Proton membrane reactor for methane upgrading	Fe@SiO_2_±SrCe_0.8_Zr_0.2_O_3–*δ*_ (Methane side) | SrCe_0.7_Zr_0.2_Eu_0.1_O_3–*δ*_ (Membrane)	950	1	N/A		90% CH_4_/Ar	2.1 (Methane)	>90 (C2 products)	50 h, 1030 °C, and 1 atm	[155]
		1000					8.3 (Methane)			
		1030					15.5 (Methane)			
		1050					23.7 (Methane)			
Proton membrane reactor for methane upgrading	Mo/H‐ZSM5 (Methane side) | SrCe_0.95_Yb_0.05_O_3‐*α*_ (Membrane)	677	1	N/A		85% CH_4_/Ar+CO_2_	6.5–8.5 (Methane)	72 (C6‐C12 products)	25 h, 720 °C, and 1 atm	[153]

**Figure 18:**
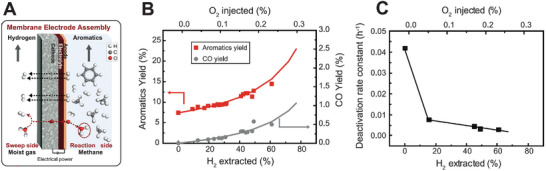
Converting CH_4_ to aromatics in PCECs. Reproduced with permission.^[^ ^23^ ^]^ Copyright 2016, AAAS. A) A Tubular reactor with the proton‐conducting membrane. B) The aromatic yield and CO yield as a function of H_2_ extracted and O_2_ injected. C) Deactivation rate constant as a function of H_2_ extracted and O_2_ injected.

**Figure 19:**
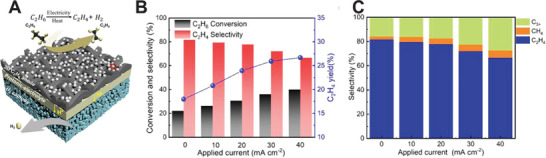
Converting C_2_H_6_ to light olefins in PCECs. Reproduced with permission.^[^ ^157^ ^]^ Copyright 2021, American Chemistry Society. A) Planar reactor with a proton‐conducting membrane for converting C_2_H_6_ to light olefins. B) Conversion of C_2_H_6_ and C_2_H_4_ yield as a function of applied current density. C) Product selectivity as a function of applied current density.

**Figure 20:**
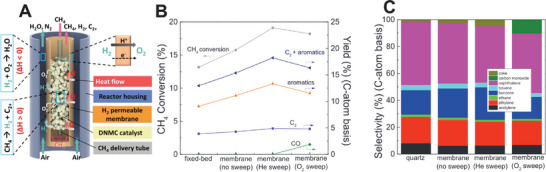
Direct nonoxidative methane conversion (DNMC) transforms CH_4_ to higher (C2+) hydrocarbons and H_2_ in an MIEC membrane. Reproduced with permission.^[^ ^158^ ^]^ Copyright 2021, Wiley‐VCH. A) Tubular reactor with a MIEC as the membrane. B) CH_4_ conversion and product yield achieved in a fixed‐bed reactor and the tubular membrane reactor using different gas to seep the H_2_. C) Product selectivity achieved in a fixed‐bed reactor and the tubular membrane reactor using different gas to seep the H_2_.

**Figures** **18**, **19**, **20** present the most recent demonstrations of converting low‐cost alkane molecules, including CH_4_ and C_2_H_6_, to aromatics and light olefins. As shown in Figure 18, J. M. Serra and C. Kjølseth et al. have focused on employing tubular PCECs with proton‐conducting membranes to upgrade methane in PCECs.^[^ ^23^ ^]^ The electrolyte membrane they employed exhibits mixed protonic and oxygen‐ion conduction. Under an external power, as shown in Figure 16A, H_2_ extraction and minor oxygen injection are simultaneously achieved. By using PCECs for methane aromatization, the methane conversion and aromatic yield have been enhanced. Furthermore, the coking has been significantly inhibited, which enables durable operation. It should also be recognized the operating temperature of methane aromatization in PCECs is around 200 °C lower than methane aromatization in conventional packed bed reactors, validating the benefits of using PCECs for upgrading methane to aromatics.^[^ ^138^ ^]^

Ding et al. have developed a planar PCEC for upgrading C_2_H_6_ to C_2_H_4_ and other light olefins.^[^ ^25^, ^157^ ^]^ Figure 19A displays the reactor designed for C_2_H_6_ dehydrogenation and H_2_ separation, which enhances C_2_H_6_ conversion. Upon applying a current to extract the H_2_, C_2_H_6_ conversion is enhanced while the selectivity to C_2_H_4_ is reduced, which is attributed to the increased selectivity to C3+ and CH_4_. This work has validated that extracting H_2_ from the C_2_H_6_ stream can shift the reaction equilibrium towards a higher conversion. Additionally, the extent of H_2_ extraction affects product selectivity. Increasing the current density tends to increase the number of carbon atoms in hydrocarbon molecules. However, this work does not identify if the C3+ compounds are alkanes or olefins. As light olefins have a higher economic value than alkanes, it is expected that increasing the current density could lead to a higher yield of C3+ light olefins.

The reactors that are shown in Figures 18A and 19A consume electrical energy to drive the H_2_ separation.^[^ ^157^, ^158^ ^]^ The reactor equipped with a mixed ionic and electronic conductor can extract H_2_ and inject O_2_, which is driven by the chemical potential. As shown in Figure 20, Wachsman et al. have demonstrated intriguing results using a mixed protonic and electronic conductor, SrCe_0.7_Zr_0.2_Eu_0.1_O_3‐□_, as the electrolyte membrane and integrating the rationally designed methane aromatization catalyst with tubular reactors.^[^ ^78^, ^155^, ^156^ ^]^ Without external applied current/voltage, the reactor functions as a hydrogen permeation membrane reactor, which extracts H_2_ from the CH_4_ side, shifting the reaction toward a higher CH_4_ conversion. This membrane reactor leads to higher CH_4_ conversion and product yield than the conventional fixed‐bed reactor. Moreover, the sweeping gas to remove the H_2_ flux affects the performance. Using an inert gas, such as helium, results in the highest CH_4_ conversion and aromatics yield. However, as both sides of this reactor are under a reducing atmosphere, the H_2_ permeation exacerbates the coking as no oxygen injection is simultaneously achieved. Using oxygen as the sweep gas changes the membrane as a mixed proton, oxygen‐ion, and electronic conductor, which concurrently extracts H_2_ and injects O_2_. As shown in Figure 20, despite the oxygen injection leading to the production of CO, both CH_4_ conversion and aromatics yield are higher than that achieved in conventional fixed‐bed reactors. Furthermore, using oxygen as the sweep gas mitigates coking and achieves durable operation. However, as the H_2_ permeation is thermally activated, the operating temperature of reactors shown in Figure 16B is higher than that of PCECs displayed in Figure 16A.

These works present new scenarios of producing value‐added chemicals (aromatics and light olefins) from natural gas, which enhances the onsite utilization of natural gas and reduces emissions, offering an economically viable and environmentally benign alternative to the conventional conversion of natural gas.

### 4.3 Challenges and Opportunities for Upgrading Methane in PCECs

Upgrading natural gas in PCECs also faces some challenges. These challenges and corresponding strategies are outlined as follows:

There is a limited catalyst that has been deliberately designed for PCECs.^[^ ^159^ ^]^ The current methane aromatization catalysts integrated with PCECs are developed for thermochemical reactors, which generally operate at a higher temperature than PCECs. Furthermore, the chemical compatibility of methane aromatization catalysts with proton‐conducting ceramic has not been studied. It is therefore suggested to rationally design methane aromatization catalysts that are chemically and physically compatible with PCECs. Furthermore, if the methane aromatization catalyst simultaneously exhibits excellent conductivity and catalytic activity, the catalyst can be directly applied to PCECs as an electrode or catalytic layer, spatially expanding the active electrochemical reaction sites and simplifying PCEC manufacturing.

The current PCEC configuration is limited to the tubular geometry, which allows to directly fill the PCEC tube with methane aromatization catalysts. However, the tubular PCEC requires more complicated manufacturing processes. Therefore, developing PCECs and catalysts that could also be integrated with planar PCEC cells is essential for its future development, further enhancing its versatility for upgrading natural gas to high‐value chemicals.

## 5 Prospects and Opportunities

The research and development of PCECs for synthesizing chemicals are under enlargement. Electrochemical approaches for chemical production are being proven to be economical, sustainable, and environmentally benign as they can utilize fossil fuels more efficiently or fully avoid the utilization of fossil fuels while using renewable power and abundant feedstocks such as nitrogen and water. Producing chemicals in PCECs on the other hand opens an attractive avenue for chemical energy storage as PCECs interconvert the electrical energy and chemical energy.

### 5.1 Interdependency between Chemical Reactions

Thinking beyond synthesizing chemicals via a single reaction, intensifying two or more reactions in one PCEC to produce more chemicals can enhance the energy efficiency, reduce capital cost, and improve economic benefits. For example, N_2_ or CO_2_ reduction PCEC can be integrated with the CH_4_ dehydroaromatization reactor to utilize the H_2_ produced via CH_4_ conversion. N_2_ or CO_2_ reduction electrodes can be applied as the negative electrodes of the CH_4_ conversion reactor (Table 1); thus, H_2_ will directly reduce N_2_ to ammonia or convert CO_2_ to value‐added chemicals. This process intensified reactor increases the hydrogen utilization and reduces the carbon footprint of producing ammonia, carbon monoxide, and hydrocarbons.

Due to the higher operating temperature (300–500 °C) of PCECs than low‐temperature (<100 °C) electrochemical devices, HER is favorable. Both CO_2_ reduction and N_2_ reduction in PCECs suffer from high Faradaic selectivity toward H_2_. Additionally, the high operating temperature leads to thermally activated decomposition or conversion of the chemicals produced in PCECs. For example, at 500 °C, ammonia can be cracked to H_2_ and N_2_, which further reduces the ammonia production rate and Faradaic selectivity. Therefore, some common strategies can be established to suppress HER for both CO_2_ reduction and N_2_ reduction. For example, using Ni‐free negative electrode to inhibit hydrogen production.

### 5.2 Impacts of Renewable Electricity Cost on the Economic Viability of Producing Chemicals in PCECs

The economic viability of producing chemicals in PCECs depends on both capital and operational costs. The reduction in electricity cost, especially the renewable electricity cost, is essential for facilitating the future implementation of PCECs for synthesizing chemicals in the real world. In the last decade, with the growing deployment of wind‐ and solar‐based renewable power plants, the renewable electricity cost has been drastically reduced. It is expected that renewable electricity will become more widely available. However, the global power demand is also growing, and electricity cost is determined by the demand and supply. Therefore, to accurately evaluate the economic viability of producing chemicals in PCECs, it is suggested to conduct a comprehensive techno‐economic analysis to quantitively determine the chemical production cost in PCECs and compare it with conventional manufacturing processes.

### 5.3 Electrodes and Scaleup

Future work should primarily focus on the development of advanced electrodes and catalysts for PCECs, which could significantly improve the feedstock conversion, reduce the electrical energy consumption, enhance the product yield, and modulate the selectivity toward a certain chemical. Nowadays, most efforts have been devoted to synthesizing chemicals in lab‐scale PCECs that exhibit an active area of up to 5 cm^2^, which is far from practical applications. The scaleup and commercialization of PCECs for synthesizing chemicals also lag far behind other technologies. Therefore, future studies should also be centered on designing, fabricating, and characterizing PCERs at the bench scale or commercially relevant scale.

### 5.4 Fundamental Understanding of the Reactions at Electrodes

Substantial increasing efforts have been devoted to validating the concepts of using PCECs for chemical production. Although compelling performances have been demonstrated for ammonia synthesis, CO_2_ reduction, and natural gas conversion, there is a lack of fundamental understanding of the electrochemical reactions and reaction pathways. Therefore, it is necessary to design and build the experimental tools to assist in probing mechanisms of nitrogen reduction reaction, CO_2_ reduction reaction, and methane aromatization in PCECs. A deeper understanding of the reaction mechanisms could be of great benefit to rationally designing electrodes and catalysts for PCECs.

### 5.5 Optimize Positive Electrodes to Improve the Feedstock Flexibility

The configurations of PCECs employed for producing chemicals vary with feedstocks and products, which limits the flexibility and versatility of PCECs. For example, the positive electrodes of PCECs for CO_2_ reduction should be modified to use different reactants delivered to the positive electrode. With a positive electrode that is active for the oxidation of various feedstocks, PCECs will be more capable of converting broader reactants to high‐value chemicals.

### 5.6 Operating Conditions

Finally, we have recognized that operating conditions, including temperature, atmosphere, current density or voltage, and pressure, can affect conversion, selectivity, and yield. All these factors are intertwined to impact the PCEC performances, suggesting a detailed computational system can be developed to control and optimize the processes, aiming to further enhance the economic viability of producing chemicals in PCECs.

## 6 Conclusion

In this review, we have focused on summarizing and analyzing the recent demonstrations of PCECs for synthesizing chemicals, which include ammonia synthesis, reducing CO_2_ to fuels, and upgrading natural gas to high‐value chemicals. For each application, we have provided the background and fundamental understanding of materials, with a particular focus on the electrode where the essential reactions occur. We have also summarized the corresponding reactions and thermodynamics. Moreover, we have highlighted the promising demonstrations of PCECs. For each application, the challenges have been summarized. The strategies to address these challenges, enhance performance, and improve stability have been proposed. Overall, this review aims to provide comprehensive guidance for the researchers to design and optimize PCECs for synthesizing sustainable chemicals and fuels.

## Conflict of Interest

The authors declare no conflict of interest.
